# Prokaryotic community structure and auxin biosynthesis in early developmental stages of farmed Atlantic Nori (*Porphyra* spp.)

**DOI:** 10.3389/fmicb.2025.1750184

**Published:** 2026-01-21

**Authors:** Francisco Cortez, Enrico Nanetti, Guilherme Chaves, André C. Pereira, Madalena C. Mendes, Inês Oliveira, Daniela Leuzzi, Helena Abreu, Margarida Martins, Ricardo B. Leite, Tina Keller-Costa, Rodrigo Costa

**Affiliations:** 1 iBB–Institute for Bioengineering and Biosciences and i4HB–Institute for Health and Bioeconomy, Instituto Superior Técnico, Lisboa, Portugal; 2 Department of Bioengineering, Instituto Superior Técnico, Lisboa, Portugal; 3 Unit of Microbiome Science and Biotechnology, Department of Pharmacy and Biotechnology, University of Bologna, Bologna, Italy; 4 GreenCoLab, Associação Oceano Verde, Universidade do Algarve, Faro, Portugal; 5 ALGAplus–Production and Trading of Seaweed and Derived Products S.A., PCI–Creative Science Park, Ílhavo, Portugal; 6 Department of Chemistry, Centre for Environmental and Marine Studies, University of Aveiro, Aveiro, Portugal; 7 Fano Marine Center, The Inter-Institute Center for Research on Marine Biodiversity, Resources and Biotechnologies, Fano, Italy; 8 Bantry Marine Research Station, Cork, Ireland; 9 Instituto Gulbenkian de Ciência, Oeiras, Portugal

**Keywords:** 16S rRNA gene sequencing, algal morphogenesis, algal-microbiome interactions, aquaculture, Atlantic Nori cultivation, macroalgae

## Abstract

**Introduction:**

Algal-microbiome interactions are considered pivotal for host health and development. Current understanding of the diversity and function of algal-associated microorganisms in aquaculture settings remains limited, preventing the development of microbiome-based solutions for sustainable algal growth.

**Methods:**

We employed cultivation-dependent and -independent approaches to determine the structure of bacterial communities associated with farmed Atlantic Nori (*Porphyra dioica* and *Porphyra umbilicalis*) at early developmental stages. 16S rRNA gene amplicon sequencing and cultivation of bacterial symbionts were performed for algal and culturing water samples harvested from indoor photobioreactors at stages S1 (conchocelis cultures growing vegetatively), S2 (conchosporangia), and S3 (young blades).

**Results:**

The phyla *Pseudomonadota* (*Alpha*- and *Gammaproteobacteria* classes) and *Bacteroidota* were dominant in algal samples, followed by *Planctomycetota*, *Actinobacteriota,* and *Verrucomicrobiota*. At the phylotype level, these communities were highly structured throughout the host’s life cycle. Uncultivated lineages Sva0996 (*Actinomycetota*), OM190 (*Planctomycetota*), Pir4 (*Planctomycetota*), and the genera *Blastopirellula*, *Algoriphagus*, *Hyphomonas*, and *Marinobacter*, among others, were enriched in algal samples and presented significantly different abundances across developmental stages. In some cases (e.g., genera *Aquimarina*, *Sulfitobacter*, *Maribacter*, and *Nonlabens*), those changes were also observed in culturing water. Moreover, the genera *Ensifer* (*Rhizobiaceae*), *Paraglaciecola* (*Alteromonadaceae*), and the uncultivated lineages DEV007 (*Verrucomicrobiota*) and Pir4 (*Planctomycetota*) were consistently present in *P. dioica* and *P. umbilicalis* samples at multiple developmental stages. Several *Porphyra*-associated bacterial genera and putative novel species, mostly belonging to the families *Roseobacteraceae*, *Flavobacteriaceae*, and *Alteromonadaceae* were identified via cultivation. Many cultured members of the *Porphyra* microbiome produced the growth-promoting hormone auxin, particularly those belonging to the genera *Alteromonas*, *Marinobacter, Sulfitobacter, Leucothrix,* and *Roseovarius*.

**Discussion:**

This study unveils complex, phylogenetically distinct, and temporally structured bacterial communities possessing algal morphogenesis-inducing capacities during early developmental stages of *Porphyra* spp., highlighting the potential of microbiome-based interventions for sustainable growth of marine algae in aquaculture.

## 1 Introduction

Macroalgae (referred to as seaweeds in industry, trade and policy) are photosynthetic, multicellular eukaryotes which play pivotal roles in aquatic habitats as primary producers and ecosystem engineers. In addition to their ecological importance, the exploitation of macroalgae as food, animal feed, fertilizers, cosmetics and nutraceuticals (Araújo et al., 2021; Zhang B. et al., 2022) has increased markedly over recent decades, with Asia leading global production and export of macroalgal products (Abdul Malik et al., 2020; FAO, 2024). Macroalgae aquaculture is a diverse and versatile industry, often considered more sustainable and productive than conventional agriculture (Araújo et al., 2021; Zhang L. et al., 2022). To meet rising demand across various sectors, aquaculture production has expanded significantly, now greatly exceeding the harvest of wild macroalgae, except in Western regions such as Europe (FAO, 2024). However, macroalgal aquaculture remains restricted to a small number of species for which complex but well-established cultivation techniques exist (FAO, 2021; Mendes et al., 2022; Pereira and Yarish, 2008).

Foliose Bangiales (*Porphyra sensu lato*) is a group of red macroalgae in the phylum Rhodophyta, which includes the economically important ‘laver’ or ‘nori’. This group comprises more than 100 species now distributed across several genera (Sutherland et al., 2011). These algae are valued for their use as food, nutraceuticals, and cosmetics (Van Jesse et al., 2022), owing to their high content of carbohydrates, proteins, vitamins, minerals, and unique bioactive compounds such as the sulphated polysaccharide porphyrin, which has health-promoting potential (Kim et al., 2016; Pereira and Yarish, 2008; Wells et al., 2017). Despite their significance, aquaculture of commercially valuable *Porphyra/Pyropia* species remains uncommon outside Asia (FAO, 2021). To increase yields and reduce production time, costs, and vulnerability to environmental stresses, alternative cultivation techniques need to be optimized and implemented (Cottier-Cook et al., 2022; Pereira et al., 2024; Zhang L. et al., 2022). The complex and heteromorphic life cycle that characterizes species within this group, alternating between a haploid gametophytic phase and a diploid sporophytic phase, makes *Porphyra* cultivation challenging, especially in the context of intensive biomass production (Baweja et al., 2016; Blouin et al., 2007; Royer, 2017). Overcoming these hurdles may be possible by advancing our understanding of the complex microbial consortia associated with *Porphyra* species (Li et al., 2023; Ramanan et al., 2016). The macroalgal microbiome comprises a diverse and dynamic community of microorganisms which include bacteria, archaea, viruses, fungi, and other microeukaryotes, that colonize macroalgal surfaces (Abdul Malik et al., 2020). Although algal microbiomes can vary with geographical location and seasonality (Cai et al., 2023; Davis et al., 2023; Nousias and Montesanto, 2021), they are often reported to be species-specific (Goecke et al., 2013; Hollants et al., 2013; Ramanan et al., 2016), to shift throughout the life cycle and developmental stages (Cai et al., 2023; Davis et al., 2023; van der Loos et al., 2024) and to be vertically transmitted between generations (Syukur et al., 2024). Such a “microbial gardening” process suggests a significant interdependence between the algal host and its microbiome, the disruption of which can lead to disease, pathogenic colonization (Abdul Malik et al., 2020; Mohammed Riyaz et al., 2021; Tasdemir et al., 2024) or even incomplete morphogenesis. Indeed, the growth and morphogenesis of *Porphyra*, as well as other algae (e.g., *Ulva*), are strongly influenced by microorganisms (Egan et al., 2006; Marshall et al., 2006; Provasoli and Pintner, 1980). For example, axenic cultures of the closely related *Pyropia yezoensis* have been shown to lose normal morphogenetic capacities compared with individuals with a microbiome (Yamazaki et al., 1998). Furthermore, bacterial taxa commonly associated with *Porphyra umbilicalis* (e.g., *Sulfitobacter*) have been identified as key contributors to algal growth by synthesizing morphogenetic compounds and auxin-like phytohormones (Amin et al., 2015; Aydlett, 2019; Miranda et al., 2013). Growth-promoting traits have also been reported for the genera *Maribacter* and *Roseovarius* in the sea lettuce *Ulva mutabilis*. Specifically, bacterial morphogenetic compounds, also known as morphogens, can induce rhizoid formation, cell wall development, blade cell division, and thallus elongation in this green alga (Alsufyani et al., 2020; Weiss et al., 2017). Therefore, gaining deeper knowledge about the biodiversity and functional roles of bacterial symbionts in algal health, growth, and development is likely crucial for improving and sustaining aquaculture production systems through microbiome management (Li et al., 2023; Natrah et al., 2014). This may be particularly important at early stages of algal development, when algae are typically produced in controlled nursery conditions and may be more susceptible to colonization by beneficial microbes (Li et al., 2023; van der Loos et al., 2024). However, few studies have explored microbial community structure throughout the life stages of *Porphyra* grown in controlled laboratory or aquaculture systems (Aydlett, 2019; Sunairi et al., 1995; Yamazaki et al., 1998; Zeb et al., 2024). Most species, such as *P. dioica*, remain largely neglected, whereas others, including *P. purpurea*, *Py. yezoensis*, *P. umbilicalis*, have primarily been studied in the wild (Aydlett, 2019; Duan et al., 1995; Fukunaga et al., 2009; Kim et al., 1992; Miranda et al., 2013; Nousias and Montesanto, 2021).

This study aims to provide baseline data on the structure and diversity of prokaryotic communities at early developmental stages of two *Porphyra* species, *P. dioica* and *P. umbilicalis*, maintained in an Integrated Multi-Trophic Aquaculture (IMTA) system without artificial pesticides or fertilizers. We investigated samples from the first three developmental stages (nursery phase) of both species, grown indoors in photobioreactors under controlled conditions, using both culture-dependent and 16S rRNA gene metabarcoding approaches. Culturing water samples were also analyzed using cultivation-independent methods. Our results reveal abundant, complex, and phylogenetically unique bacterial communities associated with the early developmental stages of these economically valuable red algae, and we discuss the potential roles of *Porphyra*-associated bacteria in algal health and biomass production, particularly focusing on auxin biosynthesis and algal growth-promoting capacities.

## 2 Materials and methods

### 2.1 Sample origin

Two species of farmed Atlantic Nori, genetically validated as *P. dioica* and *P. umbilicalis*, and surrounding culturing water were obtained from the facilities of the aquaculture company ALGAplus (Ílhavo, Portugal, 40°36′43″N, 8°40′43″W).^1^ Samples were collected on 9th November 2023, for *P. dioica*, and on 21st March 2024, for *P. umbilicalis*. *Porphyra* production entails five developmental stages (S1 to S5), the first three of which (S1 to S3) are cultivated in indoor nurseries under controlled stocking density, temperature, light, and aeration conditions (Supplementary Figure S1). These three stages represent the early phases of the life cycle of *Porphyra*: S1 corresponding to stock mother cultures of conchocelis growing vegetatively (climate chamber 1); S2 to conchocelis cultures where the differentiation to the conchosporangia stage takes place (climate chamber 2); and S3 to vegetatively growing young blades (climate chamber 1). Nursery cultivation occurred in batch photobioreactors of 250 mL to 4 L with autoclaved seawater from the IMTA system scaled up to 20 L in stage S3. For *P. umbilicalis*, autoclaved seawater was still used in this stage, while filtered IMTA seawater (100 μm pore size filter) was used in S3 for *P. dioica*.

### 2.2 Sampling and sample processing

For each developmental stage (S1 to S3), four independent *P. dioica* (Pdi) and *P. umbilicalis* (Pu) replicates, each consisting of 2 g (wet weight, ww) of algal biomass, were collected under a laminar flow chamber, placed into 50 mL sterile propylene tubes, and covered with culturing water. In addition, two independent replicates of culturing water (1 L per sample) from each *Porphyra* species and developmental stage were collected and stored into sterile zip lock bags. Thus, a total of 24 algal tissue samples, corresponding to 12 samples from each species (Pdi1, Pdi2, Pdi3; Pu1, Pu2, Pu3; *n* = 4 replicate samples per species – Pdi and Pu - and developmental stage – 1,2,3), as well as a total of 12 water (W) samples (S1 to S3: WPdi1, WPdi2, WPdi3; WPu1, WPu2, WPu3; *n* = 2 replicate samples per algal species and developmental stage), were collected. Samples were transported in cooling boxes to the laboratory, kept overnight at 4 °C, and then processed for cultivation-independent (16S rRNA gene metabarcoding) and cultivation-dependent procedures.

For prokaryotic taxonomic profiling by means of 16S rRNA gene sequencing (“metabarcoding”), 0.5 g ww of algal tissue from each replicate sample was weighed in sterile conditions, placed in 1.5 mL Eppendorf tubes, and stored at −80 °C until total community DNA (TC-DNA) extraction. Water samples were processed via vacuum filtration under sterile conditions using MF-Millipore (Darmstadt, Germany) membrane filters with 0.2 μm pore size, 47 mm diameter, and composed of a mixed cellulose-ester matrix to retain microbial cells. Each membrane filter was folded using sterilized tweezers and placed in 1.5 mL polypropylene sterile tubes which were stored at −80 °C until further processing. Sample processing for the cultivation of *Porphyra*-associated bacteria is explained in detail below.

### 2.3 Total community DNA extraction and next-generation sequencing of 16S rRNA gene amplicons

Total community DNA (TC-DNA) was extracted from all the algal and water samples processed for metabarcoding as explained above. Specifically, 24 *Porphyra* tissue samples (0.5 g each, four replicates per species per developmental stage as explained above) were processed using the DNeasy PowerSoil Pro Kit (Catalog no. 47014, QIAGEN, Hilden, Germany) following the manufacturer’s instructions with a minor modification. Briefly, the homogenization step was performed using a Bead Mill MAX (VWR) homogenizer with the following specifications: speed 5.50 m/s, 3 cycles of 30 s, and 30 s pause between cycles. For culturing water samples, the same homogenization was carried out after cutting, under sterile conditions, each filter into small pieces and adding them to the bead-beating, cell lysis tubes of the DNeasy PowerSoil Pro Kit. DNA was quantified using a NanoDrop ND1000 UV–Vis spectrophotometer (NanoDrop Technologies, Wilmington, DE) and a Qubit 4.0 fluorometer (Invitrogen, United States) and stored at −20 °C. TC-DNA was then sent for library preparation and next-generation sequencing (NGS) of the V4 region of the 16S rRNA gene at Instituto Gulbenkian de Ciência (IGC), Oeiras, Portugal. The 16S rRNA gene amplicon libraries were generated using EMP barcoded primers 515F (5′-GTG CCA GCM GCC GCG GTA A-3′) (Parada et al., 2016) and 806R (5′-GGA CTA CHV GGG TWT CTA AT-3′) (Apprill et al., 2015) following the Earth Microbiome Project 16S Illumina Amplicon Protocol (Apprill et al., 2015; Caporaso et al., 2012; Greg Caporaso et al., 2018; Parada et al., 2016; Walters et al., 2016), with PCR cycling conditions of 94 °C for 3 min; 35 cycles of 94 °C for 45 s, 50 °C for 60 s, and 72 °C for 90 s; and a final extension at 72 °C for 10 min. Sequencing was performed on an Illumina MiSeq platform using a 2 × 300-bp paired-end protocol with 600 cycles, according to the manufacturer’s instructions (Illumina, San Diego, CA, USA). Sequencing quality control was performed using fastQC v. 0.12.1 (Andrews, 2010).

### 2.4 Analysis of 16S rRNA gene amplicon sequencing data

Raw 16S rRNA gene sequences were processed using a pipeline combining PANDAseq v. 2.11(Masella et al., 2012) and QIIME 2 v. 2021.8.0 (Bolyen et al., 2019). The “fastq filter” function of the Usearch v. 11 algorithm (Edgar, 2010) was applied to retain high-quality reads (min/max length = 200/510 bp; tot = 202,551 high-quality reads) that were then binned into 522 Amplicon Sequence Variants (ASVs) (Supplementary Table S1) using DADA2 v. 1.18.0 (Callahan et al., 2016). Chimeric and singleton reads were automatically filtered out using DADA2. Taxonomic assignment was performed using the VSEARCH v. 2.7.0 algorithm (Rognes et al., 2016) and the SILVA database v. 138.1 (Quast et al., 2012). All sequences assigned to eukaryotes (including mitochondrial and chloroplast sequences) or unassigned at the domain level were discarded. Reported taxonomic classifications were manually curated, whenever possible, using the List of Prokaryotic Species with Standing in Nomenclature (LPSN, accessed in December 2024) (Parte et al., 2020). Rarefaction was performed to match the sample with the lowest number of prokaryotic reads for normalization across samples in the alpha diversity analyses, while beta diversity and taxonomic composition analyses were carried out using non-normalized data (see below for details). Output files originated from this pipeline (i.e., relative abundance taxonomy tables, alpha diversity tables, and beta diversity distance matrices) were uploaded to the R v. 4.2.0 software where graphical representation and statistics were performed. Taxonomic composition of the samples was represented by means of relative abundance (rel. ab.) barplots and boxplots. Alpha diversity was assessed using three different metrics, namely the number of observed ASVs (richness), Faith’s Phylogenetic Diversity (PD whole tree), and the Shannon Index. Beta diversity was assessed by Principal Coordinates Analysis (PCoA) on a matrix of Bray-Curtis distances computed between samples, using Hellinger-transformed data (square root of ASV relative abundances). All statistical analyses were performed using the R software (www.r-project.org), v. 4.2.0, with the packages “vegan” v. 2.6–4 (Oksanen et al., 2013), “microbAIDeR” v. 0.2.0 (Fabbrini and Conti, 2025), and “pairwiseAdonis” v. 0.4.1 (Martinez Arbizu, 2025). The Kruskal-Wallis test and Wilcoxon rank-sum test were used to assess significant differences in taxon relative abundances between groups and in alpha diversity distributions. Permutational analysis of variance with a pseudo-F ratio (function “adonis2” in the vegan package and function “pairwiseAdonis” in the homonymous package) was performed to determine whether differences in prokaryotic community structures at the ASV level, as observed in PCoA diagrams, were significant among sample groups (*P. dioica* vs. culturing water at stages S1 to S3 and *P. umbilicalis* vs. culturing water at stages S1 to S3). *p*-values were corrected for multiple testing using the Benjamini-Hochberg method, with an FDR ≤ 0.05 considered to be statistically significant.

### 2.5 Bacterial cultivation from *Porphyra dioica* and *Porphyra umbilicalis*

For the cultivation of aerobic, heterotrophic bacteria associated with *P. dioica* and *P. umbilicalis*, 1 g ww of algal biomass from each sample was suspended in 9 mL of sterile Calcium and Magnesium Free Artificial Sea Water (CMFASW: 27 g/L NaCl, 2.41 g/L MgSO_4_*7H_2_O, 1.90 g/L MgCl_2_*6H_2_O, 1.11 g/L CaCl_2_*2H_2_O, 0.75 g/L KCl, and 0.17 g/L NaHCO_3_), by grinding with sterile mortar and pestle, under aseptic conditions. The resulting suspension was transferred to a sterile, 15-mL falcon tube containing a small laboratory spoonful of 2-mm glass beads and vigorously vortexed for 1 min. Serial dilutions were prepared thereafter with CMFASW, up to a dilution factor of 10^−6^. Aliquots of 100 μL from the 10^−3^ to 10^−6^ dilutions were spread-plated on 1:2 diluted marine agar (MA1:2 - Difco Marine Broth 2,216, Carl Roth GmbH + Co. KG - diluted with ASW). MA 1:2 and ASW were prepared as described previously (Keller-Costa et al., 2017). All dilutions were inoculated in triplicates to enable comprehensive sampling of the existing bacteria through the three developmental stages and robust estimates of Colony Forming Units (CFUs) at each stage. MA 1:2 plates were incubated at 20 °C in a static microbiological incubator (PHC, Japan) for 4 weeks.

To estimate the average CFUs per gram of algal tissue (ww) in each developmental stage, colony counting was performed on incubation days 3, 5, 7, 11, 14, 20, and 27 in the case of *P. dioica* and on days 3, 6, 10, 14, 20, and 28 for *P. umbilicalis*. The Kruskal-Wallis test followed by a Dunn’s *post hoc* test was used to detect significant differences between CFU estimates across different developmental stages at the end of the incubation period. These tests were performed using the Past software v.4.11 (Hammer et al., 2001).

### 2.6 Bacterial isolation from *Porphyra dioica* and *Porphyra umbilicalis*

Aiming to select a high diversity of bacteria from both *Porphyra* species, colonies which showed differences in color, morphology, and time of appearance were re-streaked on MA 1:2 plates and incubated under the same conditions as mentioned above. Successive streaks were performed until purity was achieved. This procedure allowed for the creation of a rich bacterial culture collection, consisting of several different morphotypes, and including also slow-growing taxa.

Pure isolates were then transferred to 10 mL of sterile 1:2 diluted Marine Broth (MB1:2; diluted with ASW) and grown at room temperature under 50 rpm agitation in sterile T25 flasks placed on a horizontal shaker (Fisher Scientific, United States). The freshly grown liquid cultures were transferred to two 2 mL Eppendorf tubes and centrifuged for 30 min at 15 °C and 10,000 *g* (Scanspeed 1730R centrifuge, LaboGene, Denmark). After supernatants were discarded, the bacterial cell pellet from one tube was stored in glycerol-filled cryogenic vials (MB1:2 + 20% glycerol) while the second pellet was frozen for total DNA extraction. All samples were stored at −80 °C until further processing.

### 2.7 Genomic DNA extraction from bacterial isolates and sanger sequencing of the 16S rRNA gene

The Wizard genomic DNA purification kit (Catalog no. A1120, Promega Corporation, Madison, WI) was used to extract genomic DNA from all isolates obtained in this study, following the manufacturer’s instructions for bacterial cells. The genomic DNA of each isolate was quantified using NanoDrop and a Qubit 4.0 fluorometer. PCR amplification and Sanger sequencing of the 16S rRNA gene were carried out to taxonomically classify the isolates, following established procedures (Esteves et al., 2013). Briefly, PCR was carried out using bacterial universal primers F27 (5′-AGA GTT TGA TCM TGG CTC AG-3′) and R1492 (5′-TAC GGY TAC CTT GTT ACG ACT T-3′) (Weisburg et al., 1991). The PCR reaction mixture contained 1x Bioline reaction buffer, 0.2 mM dNTPs, 3.75 mM Bioline MgCl_2_, 0.1 mg mL^−1^ BSA, 2% DMSO, 0.2 μM of each primer, 1.25 U of BIOTAQ DNA Polymerase (Bioline, London, United Kingdom), and 1–3 μL of 20 ng/μL genomic DNA from the isolate. Thermal cycling during the PCR was as follows: an initial denaturation at 94 °C for 5 min, 25 cycles of 94 °C for 30 s, 56 °C for 30 s, 72 °C for 45 s and a final extension step of 72 °C for 10 min. The PCR products were examined by means of a 1.2% agarose gel electrophoresis. PCR products of the correct size (c. 1,500 bp) were purified on freshly prepared Sephadex G-50 columns (Sigma-Aldrich, Missouri, United States), according to the manufacturer’s instructions. Sanger sequencing was performed using the F27 forward primer at STAB-VIDA Lda (Caparica, Portugal).^2^

### 2.8 Taxonomic assignment of bacterial isolates

The 16S rRNA gene sequences retrieved from all bacterial isolates were manually trimmed on SeqScanner v2.0 (Applied Biosystems, Massachusetts, USA) to include only high-quality and high-confidence nucleotides and assign a taxonomic classification, up to genus level, using the SINA v.1.2.12 alignment tool of the Silva database v.138.1 (Pruesse et al., 2012). Sequences unclassified at the genus level by SILVA but showing 100% identity (0.0 E-value and 100% query coverage) to type strains of formally described species as assessed with the Basic Local Alignment Search Tool (BLAST v.2.16.0) (Camacho et al., 2009) of the US National Centre for Biotechnology Information (NCBI) were manually assigned to the corresponding genus. Taxonomic nomenclature was verified and, when necessary, manually curated using the List of Prokaryotic names with Standing in Nomenclature (LPSN) (Parte et al., 2020).

### 2.9 Phylogenetic inference of 16S rRNA genes from bacterial isolates

BLAST was used to identify closest type strains to the isolates recovered in this study using the megablast algorithm with default parameters. Trimmed, high-quality sequences, including isolate sequences and those from their closest type-strains, were aligned using ClustalW within the MEGA software v.11 (Stecher et al., 2020; Tamura et al., 2021). Next, the best evolutionary model was selected within MEGA v.11 and applied for phylogenetic inference across different datasets, either including (i) all isolates or (ii) isolates classified in the orders *Alteromonadales*, *Flavobacteriales*, and *Rhodobacterales*. Phylogenetic inferences were accomplished using the Maximum Likelihood method, 5 discrete gamma categories, partial deletion with 85% site coverage cutoff, with 1,000 bootstraps, also using MEGA v.11 (Stecher et al., 2020; Tamura et al., 2021). Type-strains from different bacterial orders were included to root the tree in each analysis (at least two taxa, or up to 10% of the total number of sequences aligned). Initial tree(s) for the heuristic search were obtained automatically by applying Neighbor-Joining and BioNJ algorithms to a matrix of pairwise distances estimated using the Maximum Composite Likelihood approach and then selecting the topology with superior log likelihood value. All trees presented were drawn to scale, with branch lengths measured in the number of substitutions per site.

To assess the relatedness between uncultivated and cultivated bacteria detected in this study, the 16S rRNA gene sequences from ASVs and bacterial isolates were aligned using ClustalW within MEGA v.11, as described above, and pairwise distances for all ASVs and isolates belonging to the same order were calculated. Pairwise distances were estimated with 85% site coverage cutoff, with 1,000 bootstraps and the evolutionary method that best applied to each dataset. 16S rRNA gene sequences with pairwise distances lower than 0.01 (> 99% identity) were considered matches. Dedicated phylogenetic inferences were then performed to showcase the relatedness between ASV and isolate sequences within *Alteromonadales*, *Rhodobacterales*, and *Flavobacteriales*, which were the most abundant *Porphyra*-associated bacterial orders.

### 2.10 Screening bacterial isolates for auxin biosynthesis

The bacterial isolates retrieved in this study were screened for their ability to synthesize the phytohormone auxin (indole acetic acid – IAA). To this end, we applied a phenotypic, colorimetric method described previously (Gang et al., 2019; Glickmann and Dessaux, 1995) and implemented for marine bacteria (Matsuda et al., 2018).

Cell suspensions were prepared by growing each strain for 48 to 72 h in 8 mL MB 1:2 at 25 °C under agitation at 170 rpm in a ZWY-200D shaking incubator (Labwit, Australia), until OD_600_ (measured using a U-2000 spectrophotometer - Hitachi, Japan) reached at least 0.4. The cultures were then standardized to 0.1 OD_600_ and 0.8 mL were transferred to sterile, 15-mL polypropylene tubes containing 8 mL MB 1:2 supplemented with tryptophan 1 g/L (MB 1:2 + Trp), as this amino acid is known to induce IAA production in bacteria, being the most common precursor for its biosynthesis (Patten and Glick, 2002; Tang et al., 2023). Incubation was performed in the dark at 25 °C and 170 rpm. Every 24 h, for 4 days, 0.6 mL were sampled from each culture and centrifuged for 5 min at 16,278 *g* in a Scanspeed 1730R centrifuge (LaboGene, Denmark). Then, 0.5 mL of the resulting supernatant were tested for the presence of IAA by addition of an equal volume of Salkowski reagent (12 g/L FeCl_3_ in 429 mL/L H_2_SO_4_). Still in the dark, the mixture was gently vortexed and incubated at 30 °C for 30 min, after which the mixtures’ absorbance at 530 nm (Abs_530_) was measured in a U-2000 spectrophotometer (Hitachi, Japan). All solutions with Abs_530_ surpassing the method’s Limit of Detection (LOD), established with a calibration curve constructed with synthetic IAA (Sigma-Aldrich, Germany), were considered positive.

## 3 Results

### 3.1 Cultivation-independent analysis of prokaryotic communities in *Porphyra* spp.

#### 3.1.1 Dataset overview

In this study, 202,551 quality-filtered 16S rRNA gene reads, encompassing 522 ASVs in total, were obtained from 36 TC-DNA samples after discarding chimeras and singletons among raw reads and further filtering out reads non-assigned at the domain level (0.1% of reads), chloroplasts (61.9%), and mitochondria (2.5%) (Supplementary Table S1). Of the retained, high-quality reads, 28,560 were generated from *Porphyra dioica* samples (*N* = 12, 151 ASVs in total), 24,223 from *P. umbilicalis* samples (*N* = 12, 176 ASVs in total), and 149,768 from culturing water (*N* = 12, 334 ASVs in total). The number of high-quality reads ranged from 433 for *P. umbilicalis* sample Pu3_a (stage S3) to 15,439 for *P. dioica* culturing water sample WPdi1_b (stage S1) (Supplementary Table S1). Rarefaction curves suggested that the ASV diversity in each sample was well covered by the sequencing effort employed in this study (Supplementary Figure S2).

#### 3.1.2 Taxonomic composition at high hierarchical ranks

**Figure 1:**
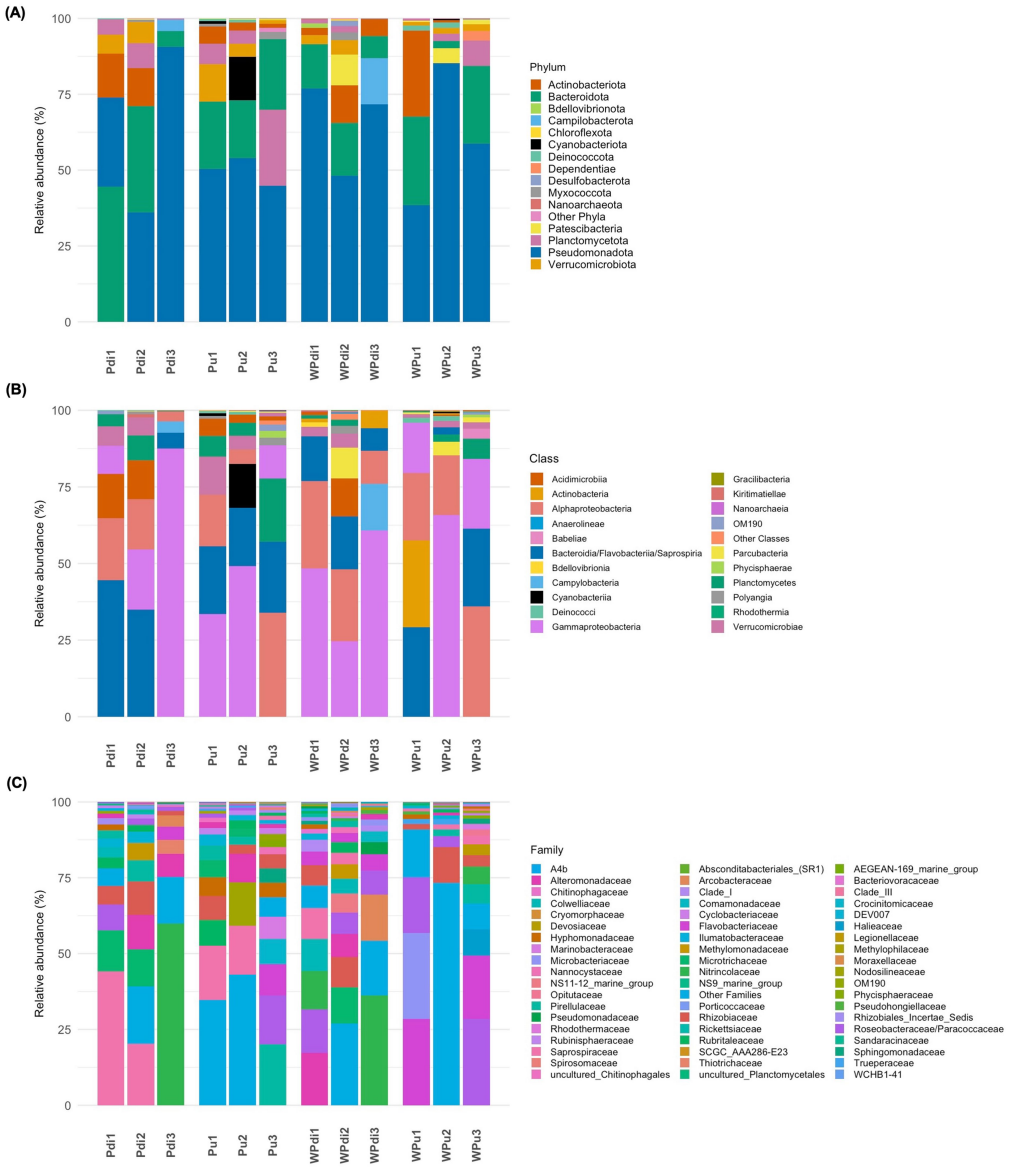
Taxonomic composition of the *Porphyra* and culturing water prokaryotic community. Bar plots summarizing the NGS-based phylum **(A)**, class **(B)**, and family-level **(C)** prokaryotic composition of *Porphyra dioica*, *Porphyra umbilicalis*, and culturing water prokaryotic communties across developmental stages. Algal species and developmental stage are reported below each bar (Pdi for *P. dioica*; Pu for *P. umbilicalis*; W for water; numbers 1–3 represent developmental stages; each bar represents average relative abundances recorded across four – algae - and two - water - independent replicate samples). Taxa legends are presented next to each plot. Only taxa with relative abundance >0.5% in at least one sample are shown. In panel 1B, the entry Bacteroidia/Flavobacteriia/Saprospiria includes reads assigned by SILVA to *Bacteroidia* but belonging to the classes *Bacteroidia*, *Flavobacteriia* and *Saprospira* according to the current validly published nomenclature.

*Pseudomonadota* and *Bacteroidota* were the dominant phyla in both algal species (Figure 1A; Supplementary Table S2). In *P. dioica*, *Pseudomonadota* showed increasing relative abundance values from S1 (mean rel. ab. ± standard deviation, 29.4% ± 11.4%) to S3 (90.7% ± 4.0%), while *Bacteroidota* displayed the opposite trend (values ranging from 44.5% ± 15.5% in S1 to 5.1% ± 1.5% in S3). Conversely, the abundance of these two phyla remained relatively constant across the developmental stages of *P. umbilicalis*, except for *Pseudomonadota* in S3 which decreased in abundance compared to the other stages. *Actinobacteriota*, *Planctomycetota*, and *Verrucomicrobiota* were detected as subdominant phyla for both *Porphyra* species. No representatives of these phyla were retrieved in our culture collection (see section below). The prokaryotic composition in culturing water partly mirrored that of the corresponding algal samples. *Pseudomonadota* and *Bacteroidota* remained the dominant phyla, despite marked abundance shifts across developmental stages (Supplementary Table S2). Relevant differences between algae and water were observed for taxa such as *Actinobacteriota*, *Campylobacterota*, *Cyanobacteriota*, and *Planctomycetota*, which were enriched in the algal samples. The archaeal domain (phylum *Nanoarchaeota*) was only observed in low abundance (mean: 0.3%) in *P. dioica* culturing water at stage S1.

*Alphaproteobacteria*, *Gammaproteobacteria*, and *Bacteroidia/Flavobacteriia* were detected as the dominant classes across all samples and developmental stages (Figure 1B; Supplementary Table S3), with relative abundance patterns somehow reflecting those of the corresponding phyla. *Planctomycetes* showed the highest abundance in *P. umbilicalis* at stage S3 (20.7% ± 11.3%), while *Verrucomicrobiae* was more represented at stage S1 for both algal species (6.3% ± 2.2% in *P. dioica*; 12.4% ± 2.5% in *P. umbilicalis*). Notably, *Actinobacteria* was only detected in culturing water, with highest abundance (28.3%) in *P. umbilicalis* rearing water at stage S1, and *Campylobacteria* was only detected in *P. dioica*, and corresponding water, at stage S3.

Several bacterial families displayed markedly different abundances in relation to sample type/developmental stage (Figure 1C; Supplementary Table S4). Overall, the most represented families in our dataset included *Saprospiraceae*, *Microtrichaceae*, *Flavobacteriaceae*, *Pirellulaceae*, *Rhizobiaceae*, *Alteromonadaceae*, and *Roseobacteraceae*/*Paracoccaceae*. Notably, the relative abundance of some families, e.g., *Colwelliaceae*, *Nitrincolaceae*, and *Saprospiraceae* in *Porphyra* samples seemed to be related, at least in part, to the microbiome of corresponding culturing water samples in a developmental stage-dependent fashion.

#### 3.1.3 Alpha and beta-diversity analyses at the ASV level

**Figure 2:**
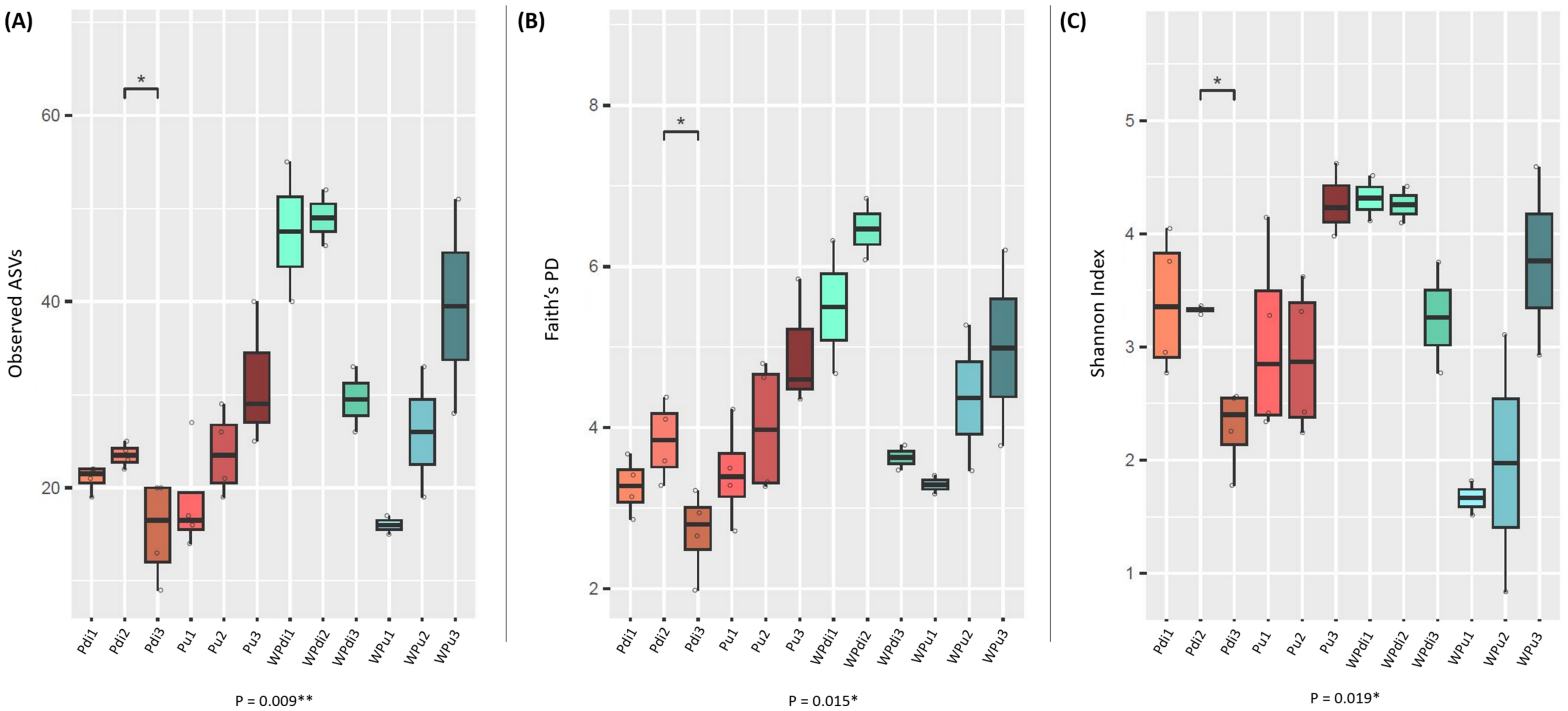
Alpha diversity analysis of prokaryotic communities associated with *Porphyra dioica*, *Porphyra umbilicalis*, and culturing water across algal developmental stages. Box-and-whisker plots showing the alpha diversity distributions of *P. dioica*, *P. umbilicalis*, and culturing water prokaryotes across developmental stages based on the number of observed ASVs **(A)**, Faith’s phylogenetic diversity **(B)**, and Shannon index **(C)** using the rarefied dataset (941 reads per sample. To visualize rarefaction curves, see Supplementary Figure S2). The horizontal bars in each box display data distributions from the first to the third quartile, with the internal bar representing the median. Whiskers show the minimum and maximum values across the distribution. The codes of each sample group are reported below each box (Pdi for *P. dioica*; Pu for *P. umbilicalis*; W for water; numbers 1–3 represent developmental stages). Results of the Kruskal-Wallis test and Wilcoxon rank-sum test controlled for multiple testing using false discovery rate (FDR) are shown in each panel; *p-value <0.05; **p-value < 0.01.

Distinct alpha diversity trends were observed for *P. dioica* and *P. umbilicalis*, as well as their corresponding culturing water, across developmental stages (Figure 2). In *P. dioica* and surrounding water, samples exhibited higher median diversity in S2 compared to S1 and S3, with S3 samples showing the lowest alpha-diversity measures. In contrast, *P. umbilicalis* and its corresponding culturing water samples displayed a progressive and significant increase in alpha-diversity from developmental stages S1 to S3, as indicated by the three alpha-diversity metrics used in the analysis: observed ASVs, Faith’s Phylogenetic Diversity, and Shannon Index (Kruskal-Wallis *p* = 0.009, 0.015, and 0.019, respectively – Figure 2). Additionally, pairwise Wilcoxon rank-sum tests highlighted significant differences in alpha-diversity between *P. dioica* samples at stages S2 and S3 across all metrics.

**Figure 3:**
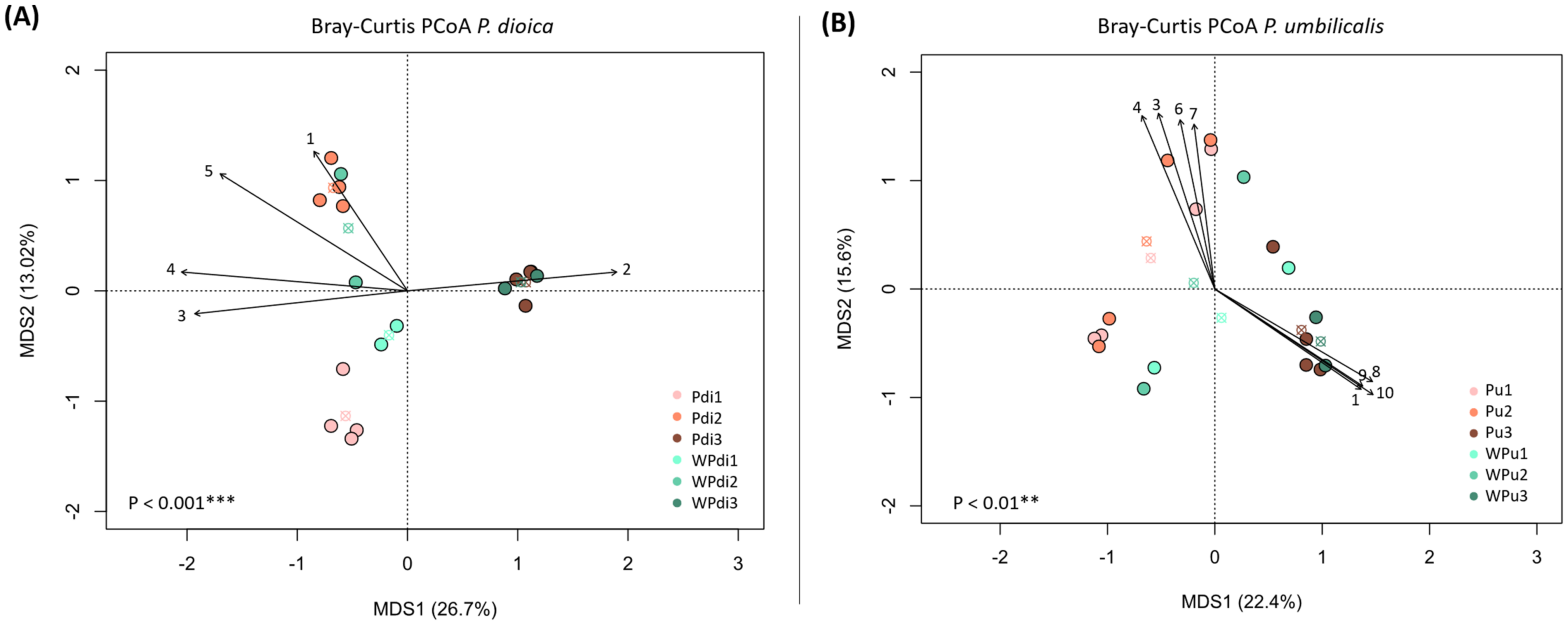
Beta diversity analysis of prokaryotic communities associated with *Porphyra dioica*, *Porphyra umbilicalis*, and culturing water across algal developmental stages. Principal coordinates analysis (PCoA) based on Hellinger-transformed Bray-Curtis distances between the microbiome of *P. dioica* **(A)** and *P. umbilicalis* **(B)**, as well as corresponding culturing water, across developmental stages. The codes of each sample group are reported in each plot (Pdi for *P. dioica*; Pu for *P. umbilicalis*; W for water; numbers 1–3 represent developmental stages). The first and second principal coordinates (MDS1 and MDS2) are plotted and the percentage of variance in the dataset explained by each axis is shown. Centroids for each group are represented as crossed circles using the corresponding color of each group. Permutation test with pseudo-F ratio; **p-value <0.01; ***p-value <0.001. Vectors represent the bacterial genera most contributing to segregations between groups, which were superimposed in the PCoA plots (function envfit of the R package vegan) considering only genera with a *p* ≤ 0.005. 1: *Blastopirellula*; 2: *Flavobacterium*; 3: Sva0996 (*Actinomycetota*); 4: Pir4_lineage (*Planctomycetota*); 5: *Ensifer*; 6: DEV007 (*Verrucomicrobiota*); 7: *Nodosilinea_PCC-7104*; 8: *Marinobacter*; 9: *OM190* (*Planctomycetota*); 10: *Sphingorhabdus*.

A pattern of segregation of prokaryotic communities according to developmental stage was also observed for both algal species and their corresponding culturing water, as revealed by ordination analysis of ASV profiles (beta-diversity analyses, Figures 3A,B). In the PCoA plot, water samples tended to cluster near the *Porphyra* samples of the same developmental stage. For instance, a somewhat large extent of variation in ASV profiles was observed among the *P. umbilicalis* water samples from stage S2, and the same trend was depicted for the corresponding algal samples from this stage (Figure 3B). Notably, dissimilarities among sample groups across developmental stages were statistically more pronounced for *P. dioica* than *P. umbilicalis* (permutation test with pseudo-F ratio). Pairwise *p*-values were significant (< 0.05) when comparing *P. dioica* samples from all different stages, and for *P. umbilicalis* when comparing S1 vs. S3 and S2 vs. S3.

#### 3.1.4 Differential distribution of bacterial genera across biotopes and algal developmental stages

**Figure 4:**
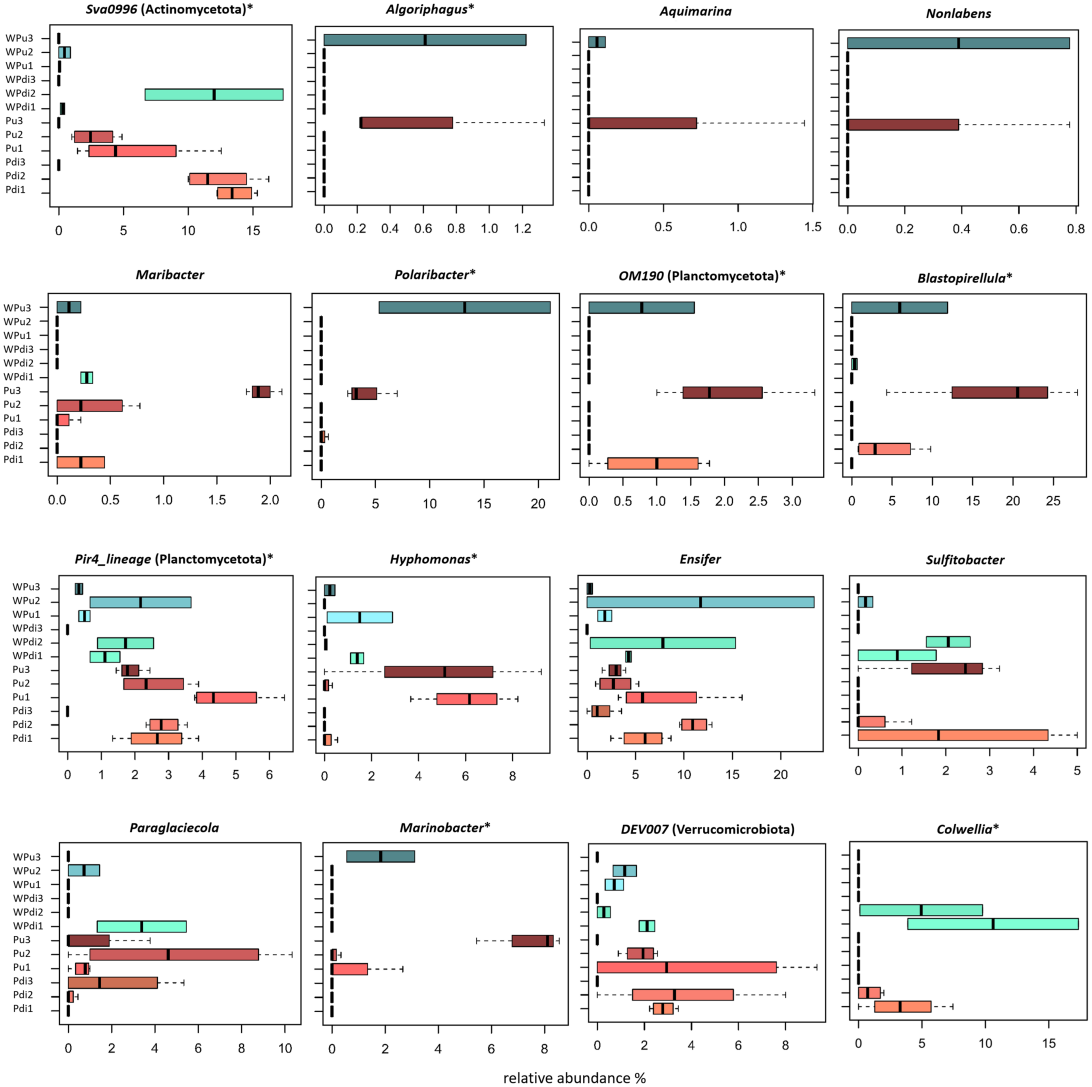
*Porphyra* and water-associated bacterial genera differently represented across developmental stages. Box-and-whisker plots showing the relative abundance distributions of select bacterial genera across the different stages. The plots shown represent bacterial genera displaying significantly different abundance distributions across stages or genera particularly abundant across several developmental stages. Legend is reported in the left part of the figure (Pdi for *P. dioica*; Pu for *P. umbilicalis*; W for water; numbers 1–3 represent developmental stages). The central box represents the distance between the 25th and 75th percentiles. The median is marked with a black line. Whiskers identify the 10th and 90th percentiles. Significant differences in abundance distributions were tested using Kruskal-Wallis coupled to a false discovery rate (FDR) correction for multiple comparisons; *p-value <0.05. Only genera with relative abundance >0.5% in at least one sample are shown.

Finally, a dedicated assessment of abundance distributions of prokaryotic genera across sample groups revealed multiple taxa displaying significant relative abundance shifts across developmental stages, host species (*P. umbilicalis* vs. *P. dioica*), and biotopes (algae vs. culturing water) (Kruskal-Wallis test controlled for multiple testing using FDR, p-value < 0.05) (Figure 4). Some of these included Sva0996 marine group, *Algoriphagus*, *Polaribacter*, OM190, *Blastopirellula*, Pir4 lineage, *Hyphomonas*, *Marinobacter*, and *Colwellia* (Figure 4). Of note, the abundance distributions of certain genera, such as *Algoriphagus*, *Polaribacter*, *Blastopirellula*, and *Colwellia*, closely mirror their relative abundances in the culturing water of the same algal species and developmental stage. Other taxa, i.e., SCGC_AAA286-E23 (*Woesearchaeales*), NS5_marine_group (*Flavobacteriales*), NS11 − 12_marine_group (*Sphingobacteriales*), *Peredibacter*, A4b, *Absconditabacteriales_*(SR1), *Paracoccus*, AEGEAN-169_marine_group (*Rhodospirillales*), *Rickettsia*, Clades Ia and III (SAR11, *Alphaproteobacteria*), *Legionella*, *Marinobacterium*, *Pseudohongiella*, and *Luteolibacter*, were only detected in culturing water, oftentimes only in one developmental stage, suggesting a depletion of these bacteria in the *Porphyra* holobiont (Supplementary Figure S3). Detailed data for all the bacterial genera detected in *Porphyra* and culturing water samples across different developmental stages (with relative abundance > 0.5% in at least one sample) are reported in Supplementary Table S5.

### 3.2 Cultivation-dependent analyses of *Porphyra*-associated bacteria

#### 3.2.1 CFU abundance and richness of colony morphotypes across *Porphyra* species and developmental stages

Due to a higher bacterial load than expected, CFU counts of *P. dioica* culturable heterotrophic bacteria for stages S1 and S2 could only be obtained at a single time point (3 days of cultivation) as subsequent days resulted in uncountable plates (CFUs > 300). This first time point, however, revealed that culturable bacterial abundance was over 1.0 × 10^6^ CFU/g of algal wet tissue. For stage S3, CFU counts reached 9.5 × 10^6^ CFU/g of algal wet tissue on the third day of incubation, gradually increasing to 1.6 × 10^7^ CFU/g of algal wet tissue after 27 days. For *P. umbilicalis*, CFU counts ranged between 3.5 × 10^5^ (stage S3) and 1.82 × 10^6^ (stage S1) CFU/g of algal wet tissue on the third day, with values steadily increasing to nearly 2.0 × 10^7^ CFU/g of algal wet tissue for stages S2 and S3 after 28 days, and reaching 5.4 × 10^7^ CFU/g for stage S1. The Kruskal-Wallis test, followed by Dunn’s *post hoc* test, indicated no significant differences in CFUs per gram of algal wet tissue between developmental stages of the same species.

#### 3.2.2 Taxonomic assignment of bacterial isolates from *Porphyra dioica* and *Porphyra umbilicalis*

**Figure 5:**
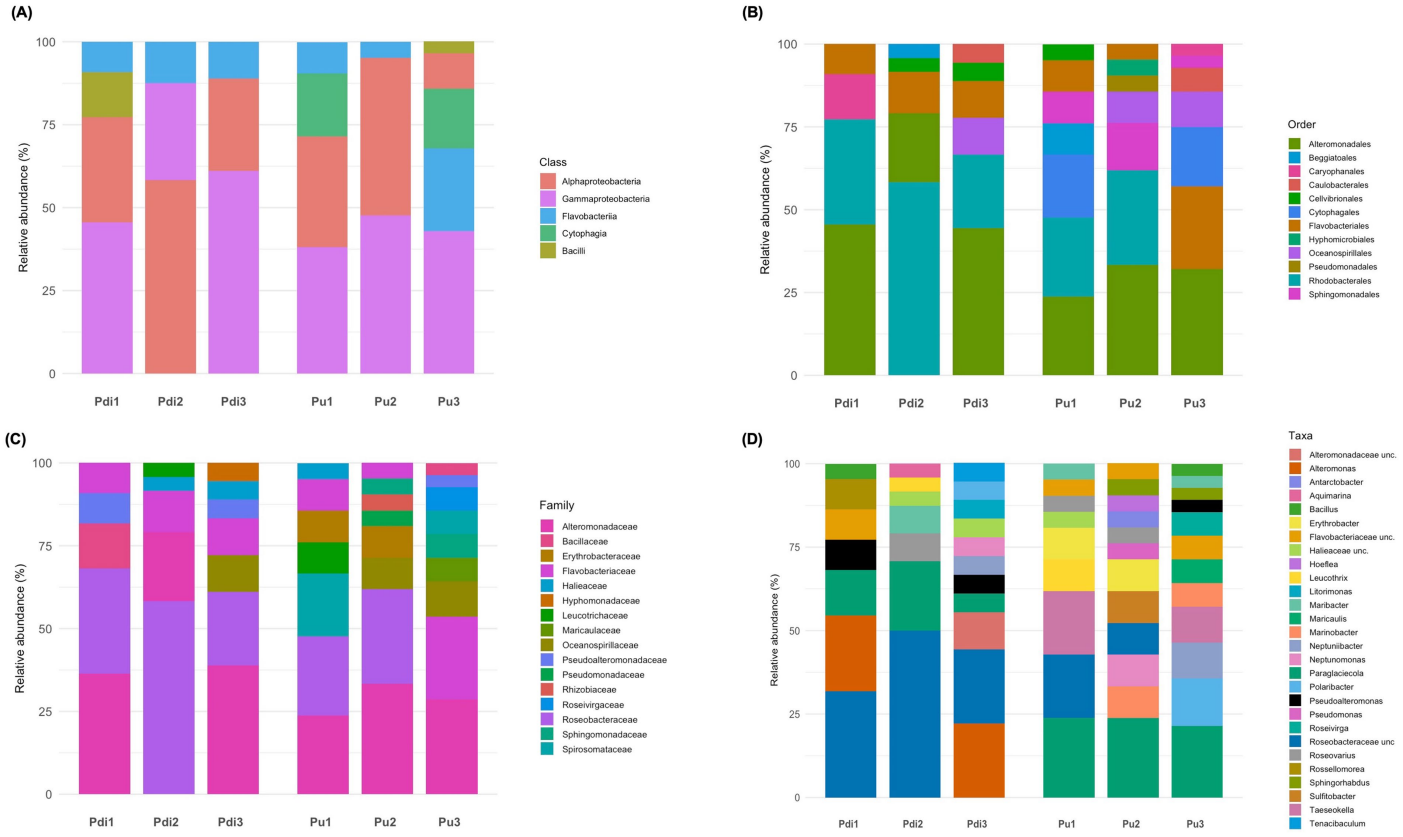
Taxonomic composition of the cultured bacterial fraction of farmed *Porphyra* species. Bar charts representing the relative abundance of classes **(A)**, orders **(B)**, families **(C)**, and genera **(D)** identified in the entire collection of bacterial isolates (*N* = 134). The relative abundance of each taxon is expressed as percentage, with the *y*-axis showing cumulative relative abundance. Algal species (*Pdi* – *Porphyra dioica*; *Pu* – *Porphyra umbilicalis*) at each developmental stage (1–3) are given below each stacked bar. Legends with taxon names are shown on the right side of each graph.

In total, more than 130 bacterial colonies were isolated, resulting in a collection of approximately 22 isolates per developmental stage and algal species. Both algal species were dominated by culturable bacteria in the phylum *Pseudomonadota* (79% of all isolates) and *Bacteroidota* (18%). Four isolates were classified as *Bacillota* (class *Bacilli*), retrieved only from *P. dioica* and *P. umbilicalis* at stages S1 and S3, respectively. *Pseudomonadota* isolates belonged to the classes *Alpha*- and *Gammaproteobacteria*, while *Bacteroidota* were assigned to the classes *Flavobacteriia* and *Cytophagia* (Figure 5A). The representation of *Pseudomonadota* peaked at stage S2, (approx. 90% of all isolates from both species), while it dropped to about 50% of the isolates in *Porphyra umbilicalis* samples at stage S3, where a sharp increase in *Bacteroidota* was observed. Isolates classified as *Bacilli*, *Cytophagia,* and *Flavobacteriia* were assigned to the orders *Caryophanales*, *Cytophagales,* and *Flavobacteriales*, respectively (Figure 5B). In contrast, *Alpha*- and *Gammaproteobacteria* isolates were classified into nine different orders (Figure 5B), with *Rhodobacterales* and *Alteromonadales* being the most abundant.

**Figure 6:**
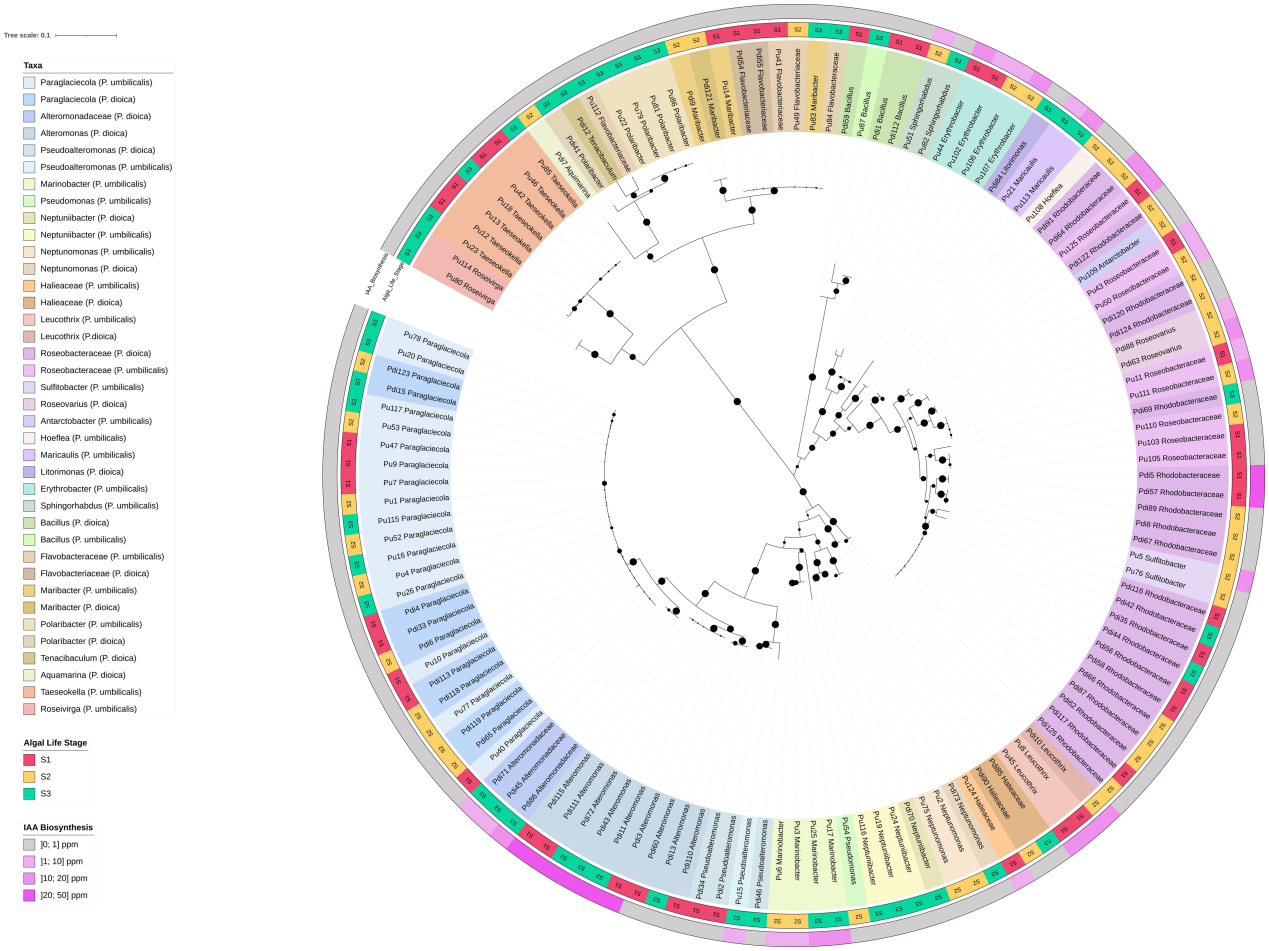
16S rRNA gene-based phylogenetic tree of bacterial isolates from *Porphyra dioica* and *Porphyra umbilicalis*. Isolates (*N* = 134) are identified with their respective strain ID as in Supplementary Table S6. Genus-level classification, whenever possible, is provided according to Silva and LPSN and isolates are colored according to their genus-level classification. Different shades within each color were used to distinguish between the provenance of the isolates according to the algal species, *Porphyra dioica* or *Porphyra umbilicalis*. The tree was constructed using the maximum likelihood method (highest log-likelihood of −10459.48) with 1,000 bootstrap repetitions, applying the Kimura 2-parameter method and discrete Gamma distributions (5 categories, +G, parameter = 0.5250) to account for evolutionary rate variation across sites (K2 + G). Branch lengths are measured in the number of substitutions per site, with the tree drawn to scale. All positions with less than 85% site coverage were eliminated, and ambiguous bases were allowed at any position using partial deletion. Bootstrap values are shown at each branch to indicate the level of statistical support. Algal developmental stage (S1–S3) is reported in the external part of the tree, in the innermost ring, while IAA production is reported in the outermost ring.

Isolates were classified into 16 families, eight of which were found in *P. dioica* and 15 in *P. umbilicalis* (Figure 5C). Eight families were common to both algal species, including the most frequent families *Roseobacteraceae*, *Alteromonadaceae*, and *Flavobactericeae*. Twenty-four bacterial genera were identified, including *Bacillus*, *Maribacter*, *Roseo*var*ius*, *Sulfitobacter*, *Paraglaciecola*, *Alteromonas*, among others (Figure 5D). Genus-level richness followed an upward trend from S1 to S3 (Figure 5D). Notably, 40 isolates could only be classified to the family level, including 26 *Roseobacteraceae* isolates that did not match formally described genera (Figure 6; Supplementary Table S6).

#### 3.2.3 Auxin biosynthesis assays

IAA production was detected in 36 strains (approx. 27% of the collection) and was observed exclusively in the *Alpha-* and *Gammaproteobacteria* (Figure 6). The most prolific producers yielded IAA concentrations between 15 and 50 ppm and belonged to the genera *Alteromonas*, *Leucothrix*, *Roseovarius*, *Sulfitobacter*, *Maricaulis*, and the *Roseobacteraceae* group.

#### 3.2.4 Phylogenetic analysis of culturable bacteria

The phylogenetic tree constructed for all isolates (Figure 6) revealed the presence of phylotypes with 100% 16S rRNA gene identity shared across multiple developmental stages and between both *Porphyra* species, including *Paraglaciecola* and certain *Roseobacteraceae* phylotypes. By integrating IAA biosynthesis data, we determined that this functional trait was conserved among some of these shared phylotypes. Specifically, identical IAA-producing phylotypes of *Roseovarius*, *Leucothrix*, and unclassified *Roseobacteraceae* were found in both algal species. Similarly, identical IAA-producing strains of *Alteromonas*, *Marinobacter*, and *Erythrobacter* were retrieved from different developmental stages of the same host (Figure 6).

Detailed phylogenetic trees of the most frequent orders (*Alteromonadales*, *Rhodobacterales,* and *Flavobacteriales*) indicated that while several isolates matched known type strains (e.g., *Pseudoalteromonas agarivorans*, *Rossellomorea arthrocnemi*, *Alteromonas stellipolaris*, *Marinobacter adhaerens*, *Leucothrix mucor*, *Pelagimonas phthalicica*, *Antarctobacter heliothermus*, *Hoeflea alexandrii*, *Roseovarius pelagicus*, and *Neptunomonas phycophila*), many others were moderately distant from type material (< 98% gene identity), suggesting they may represent novel species (Supplementary Figures S4–S6). These potential novel taxa included clusters of *Maribacter*, *Taeseokella*, and *Roseivirga*, as well as four distinct clusters of unclassified *Roseobacteraceae* primarily isolated from *P. dioica*. The closest type strains to these *Roseobacteraceae* clusters included *Sulfitobacter*, *Oceaniglobus*, *Tritonibacter*, and *Boseongicola* species.

#### 3.2.5 Comparison of culture-dependent and culture-independent community profiles

Alignment of 16S rRNA gene sequences revealed that 12.1% of the ASVs (35/289) detected in the molecular survey were captured in the culture collection (>99% identity) (Supplementary Table S7). The recovery success varied by taxonomic order: 37% of *Alteromonadales* ASVs matched isolated bacteria, followed by *Flavobacteriales* (32%) and *Rhodobacterales* (25%) (see Supplementary Figures S4, S5 for details). Conversely, 41 bacterial isolates (spanning 20 phylogenetic clusters across eight orders) did not match any ASV recovered from the algal tissue. Notably, nearly half of these isolates (19/41) matched ASVs detected in the surrounding seawater samples, including *Alteromonadales* and some *Rhodobacterales* isolates (Supplementary Table S7).

**Figure 7:**
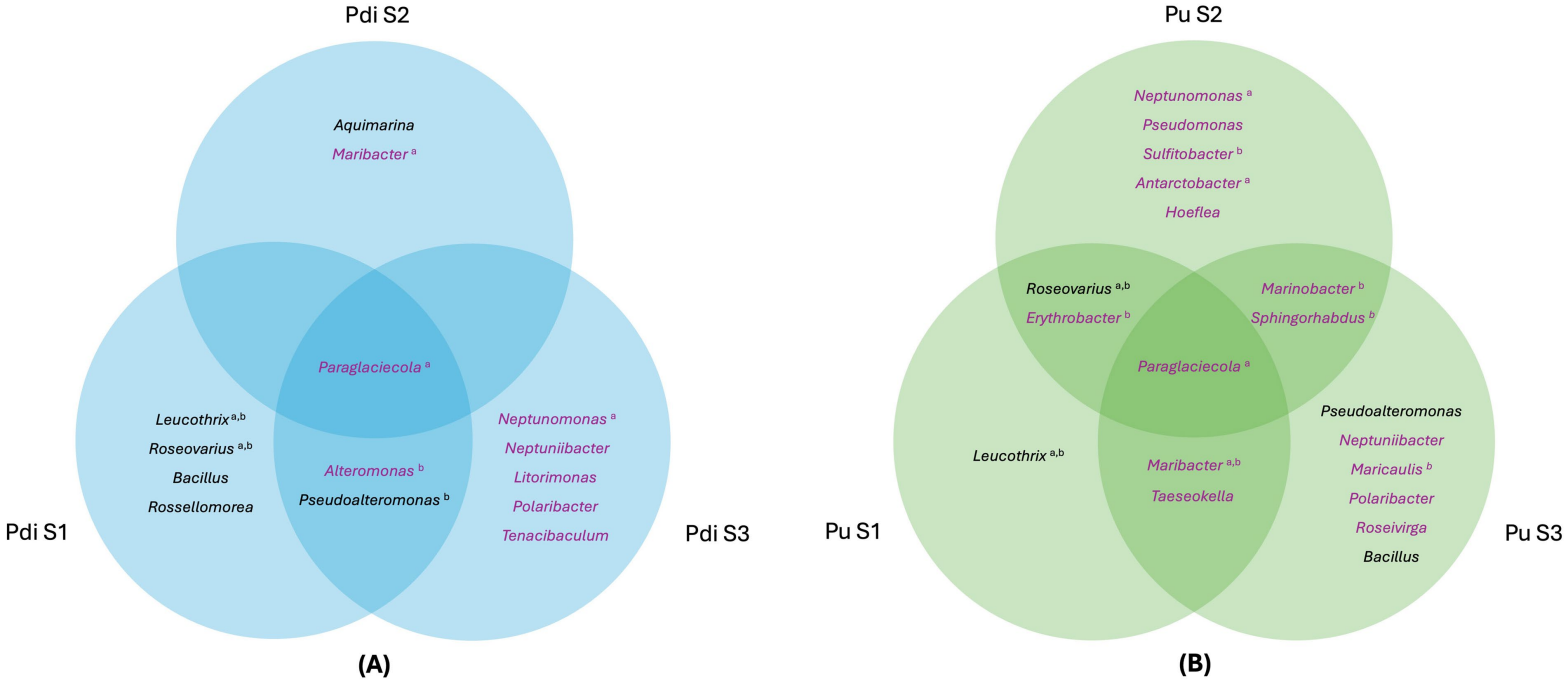
Shared and developmental stage-specific bacterial genera cultivated from *Porphyra dioica* and *Porphyra umbilicalis*. Venn diagrams illustrating bacterial genera common or specific to different developmental stages cultivated from *P. dioica* **(A)** and *P. umbilicalis* **(B)**. Genera such as *Paraglaciecola* are shared across multiple stages, highlighting their persistence throughout the life cycle, whereas genera such as *Leucothrix* (S1), *Neptunomonas* (S2), and *Polaribacter* (S3), are specific to individual stages, suggesting stage-dependent bacterial associations or associations influenced by the environment as sources of algal colonizing bacteria. Genera in which “identical” isolates (100% 16S rRNA gene identity) were identified in both *Porphyra* species are indicated ^(a)^, as are those represented by IAA-producing isolates ^(b)^. Genera in which isolates matched ASVs detected using the cultivation-independent approach (100% gene similarity) are presented in purple.

Regarding the persistence of culturable taxa, only the genus *Paraglaciecola* was isolated from all three developmental stages from both algal species (Figure 7). Although no auxin production was detected for this genus, it was well- represented in the cultivation-independent data (matching three ASVs) (Supplementary Figure S4). In contrast, genera isolated from two developmental stages such as *Alteromonas* and *Pseudoalteromonas* in *P. dioica* (Figure 7A) and *Roseovarius*, *Erythrobacter, Marinobacter, Sphingorhabdus, Maribacter*, and *Taeseokella* in *P. umbilicalis* (Figure 7B) were, except for *Maribacter* and *Taeseokella*, generally identified as prolific auxin producers (>20 ppm) (Figure 6). All isolates from these persistent genera displayed 100% 16S rRNA gene identity with detected ASVs.

## 4 Discussion

This study assessed the diversity and potential algal growth promoting capacities (i.e., auxin biosynthesis) of prokaryotic communities associated with two Atlantic Nori species (*P. dioica* and *P. umbilicalis*) at early developmental stages in an aquaculture facility. We report high bacterial densities and diversity in both species under controlled, indoor conditions. We also show clear temporal structuring of prokaryotic communities across key developmental stages involving sharp morphogenetic shifts from *conchocelis* through *conchosporangia* to young blades. The relative complexity of the described prokaryotic consortia, involving high abundance of cultivable heterotrophic bacteria and hundreds of ASVs detected in both species (151 in *P. dioica* and 176 ASVs in *P. umbilicalis*), despite using autoclaved culturing water during production, is noteworthy. This complexity may be consistent with vertical transmission (in S1) and retainment (S2 and S3) of nori bacterial symbionts from adults to offspring under controlled cultivation conditions. This hypothesis should be thoroughly tested in future studies merging targeted metagenomics and imaging approaches throughout the life cycle of Atlantic Nori, including adult blades and reproductive structures, to more specifically single out key symbionts which evolved toward a vertical transmission mode of association with their algal hosts.

### 4.1 Prokaryotic communities associated with farmed Atlantic Nori shift in structure across early developmental stages of the host

Cultivation-independent and -dependent approaches congruently revealed that the two most abundant prokaryotic phyla associated with both algae are *Pseudomonadota* and *Bacteroidota*. Such a dominance has been reported previously for wild-caught *Porphyra* spp. (Aydlett, 2019; Kim et al., 1992; Miranda et al., 2013; Nousias and Montesanto, 2021) and other macroalgae (Goecke et al., 2013; Hollants et al., 2013). As expected, the cultivation-independent approach provided a more comprehensive view, detailing not only disparities in microbial composition between samples, but also microbial shifts within dominant taxa, as well as the presence of other, less abundant phyla which were not registered through isolation either due to their low abundance or difficulty to cultivate.

Shifts in relative abundance of prokaryotic taxa were found between algal and their respective seawater samples. This suggests the establishment of a microenvironment on the algae – phycosphere – which leads to the promotion of a microbiome that is distinct from that of the surrounding seawater. Previous studies have also observed this and suggested it to be a consequence of the characteristics of algal tissue, but also due to selective pressure by the algal host through exudates to maintain certain beneficial microbial communities (Abdul Malik et al., 2020; Cai et al., 2023; Goecke et al., 2013; Hollants et al., 2013).

Most noticeably, however, we observed that the structure of *Porphyra*-associated prokaryotic communities shifted significantly across the host’s developmental stages. This was evident not only from the ordination of algal and seawater samples, as determined by ASV abundance distributions, but also from major shifts in relative abundance of certain taxa (at various taxonomic levels). Clear shifts in the composition of culturable symbionts at the genus level were also observed, with some genera being detected only in specific stages, while others persisted throughout the life cycle. In some cases, these trends were common to the molecular data at this taxonomic level, as was the case of the persistence of *Paraglaciecola* through the life cycle, or the enrichment of *Polaribacter* and *Marinobacter* at stage S3. The fact that microbial shifts seem to be more dependent on the algal developmental stage than other factors such as the microbial composition of the culturing water suggests that algal development influences the microbial communities under controlled aquaculture conditions. Although geographical location, seasonality, and the surrounding seawater are known as strong determinants of microbial diversity in wild algae (Abdul Malik et al., 2020; Davis et al., 2023; Weigel and Pfister, 2019), in the controlled environment of aquaculture production these external factors are minimized and possibly overcome by algal exudation and nutritional needs in shaping the algal microbiome. Indeed, the algal microbiota density and activity might even influence the composition of the surrounding seawater within the batch system. A previous study on farmed macroalgae also identified algal development as more influential on the microbial community than the surrounding seawater, especially in key stages of increased algal growth (Cai et al., 2023).

### 4.2 Microbiome assembly in farmed Atlantic Nori is determined by blends of functionally diverse, culturable and unculturable bacteria displaying temporal-specific and generalist patterns of host association

The microbiome of farmed Atlantic Nori emerges as a complex network of bacterial associates, some cultivated, others only detected via cultivation-independent methods. Microbial associations with the host are either stage-specific or persistent throughout its life cycle. *Paraglaciecola* stands out as the only genus isolated from all developmental stages of both algal species, while also being consistently more abundant in algal samples than in seawater samples. Known for their ability to degrade algal polysaccharides (Schultz-Johansen et al., 2018; Tanaka et al., 2022) the prevalence of *Paraglaciecola* in algal tissues likely reflects a preference for algae-derived nutrients. Whether this association affects the host positively (through carbon cycling), negatively (parasitism), or if at all (commensalism) remains unclear. However, the fact that some *Paraglaciecola* species produce vitamins of the B complex (Bayburt et al., 2024), essential for algal development, suggests a mutually beneficial association, in which the host may selectively maintain these bacteria as part of its core microbiome through the various stages of development. Surprisingly, the only other genus identified in all developmental stages of both *Porphyra* species (in this case, exclusively by cultivation-independent means) was *Ensifer*, a known symbiont of terrestrial plants with nitrogen fixation activity (Calatrava-Morales et al., 2018; León-Barrios et al., 2009) and heavy metal and organic pollutant sequestering abilities (Kumor et al., 2025; Wang et al., 2025).

*Verrumicrobiota* (DEV007) and *Planctomycetota* (Pir4_lineage) are examples of uncultivated taxa that were abundantly detected through most of the algal developmental stages (all three stages in the case of *P. umbilicalis*). Due to the inherent difficulty in cultivating these taxa (Lage and Bondoso, 2014), their roles in algal microbiomes are not yet fully understood, even though they have been extensively detected in the microbial communities of rhizosphere, other marine organisms, and extreme environments (Bergmann et al., 2011; Goecke et al., 2013; Lage and Bondoso, 2014). Nevertheless, *Verrumicrobiota* have been associated with methanotrophic activity in anoxic environments (Fenibo et al., 2023) and the degradation of complex polysaccharides (Sichert et al., 2020), while *Planctomycetota* are able to breakdown sulfated polysaccharides (Lage and Bondoso, 2014) such as porphyran (a key component in *Porphyra*). These activities might contribute to carbon cycling in the algal holobiont, roles that would be conserved through multiple stages of development. Although no negative effects on algae have been attributed to these bacterial groups, *Planctomycetota* are also known to produce bioactive compounds, have a glycoproteic holdfast, and to resist several antimicrobial compounds (Lage and Bondoso, 2014), characteristics that facilitate surface colonization and may prevent algal fouling by opportunistic microorganisms.

Besides *Paraglaciecola*, other genera from the *Alteromonadales* order were isolated from multiple stages, including *Alteromonas* and *Pseudoalteromonas* from *P. dioica* (S1 and S3), and *Marinobacter* (S2 and S3) from *P. umbilicalis*. These bacteria are also often associated with algicidal and antimicrobial activities (Abdul Malik et al., 2020; Du et al., 2023; Goecke et al., 2010; Hollants et al., 2013; Pinto et al., 2021; Ward et al., 2020), and in some cases produce growth-promoting phytohormones (Bertrand, 2022; Matthews et al., 2023). Although these traits may benefit the host through its life cycle by regulating bacterial colonizers, algal competition, and even its own growth and morphogenesis, they have often been reported as possessing deleterious or algicidal effects (Abdul Malik et al., 2020). Antimicrobial and growth-promoting activities, such as phytohormone and vitamin production, have also been reported in several *Roseobacteraceae* genera identified in multiple developmental stages. However, this family is more often associated with beneficial effects on the host. This is especially the case for *Erythrobacter*/*Qipengyuania* (isolated from stages S1 and S2) (Tang et al., 2019; Tareen et al., 2022; Zhang B. et al., 2022) and *Roseobacter* (a genus closely related to the several unclassified *Roseobacteraceae* isolates reported in this study) (Geng and Belas, 2010; Goecke et al., 2013; Spoerner et al., 2012; Wang et al., 2016). Although *Sulfitobacter* and *Roseovarius*, also part of *Roseobacteraceae*, were represented by few isolates in one or two stages, their matching ASVs were detected through all three (as were other closely related, unclassified *Roseobacteraceae*). Besides these genera also being associated with the beneficial traits mentioned above (Amin et al., 2015; Hosseini et al., 2024; Sultana et al., 2023), *Roseovarius* is particularly well known for inducing cell differentiation in *Ulva*. In fact, normal morphogenesis in *U. mutabilis* can be established through a tripartite symbiotic interaction between the host, *Roseovarius,* and *Maribacter* (Alsufyani et al., 2020; Burgunter-Delamare et al., 2024) - a *Flavobacteriales* genus extensively isolated in this study from multiple stages of both *Porphyra* species and identified in all stages of *P. umbilicalis* through the cultivation-independent approach.

Strains of the genus *Polaribacter* (also part of the order *Flavobacteriales*) also facilitate morphogenesis (Grueneberg et al., 2016), and algal spore settlement and germination through the production of Quorum Sensing metabolites and biofilms (Hollants et al., 2013). This genus was abundantly detected in a single developmental stage (S3) of both algal species through cultivation-dependent and independent approaches. This suggests a more temporally determined pattern of association with the host. In this case, the abundance of *Polaribacter* may have facilitated spore germination into young blades, which corresponds to the transition into stage S3. Other such cases where detection was exclusive to a single stage of development through both cultivation-dependent and -independent approaches include *Leucothrix* (S1), *Sphingorhabdus* (S3), *Maricaulis* (S3), and *Roseivirga* (S3). However, reports on the ecological roles of these genera on algal health or development are scarce. The biofilm formation capacities of *Maricaulis* (Abraham et al., 1999) and *Leucothrix* (Bland and Brock, 1973) are well known, but it is uncertain how these affect the host.

Assessing which of these traits are encoded in the isolated bacteria through genome analysis, or phenotypic screenings, would help elucidate their roles within the holobiont and provide deeper insights into their temporally specific or generalist associations with the host. This would not only help clarifying the factors driving the observed microbial shifts and their effect on the host but also serve as a foundation for future microbiome manipulation experiments aimed at improving algal growth and development (Li et al., 2023).

### 4.3 The culturable bacterial fraction of farmed Atlantic Nori is highly diverse and displays algal growth-promoting potential

The number of bacterial taxa isolated from both algal species represents a considerable portion of the bacterial richness detected through the cultivation-independent approach. Isolates make up over 12% of all ASVs detected in the algal samples (35/289). Similar cultivation approaches were reported by Hardoim et al. (2014) and Keller-Costa et al. (2017) to recover 10–14% and up to 61% of the bacterial richness observed in marine sponges and octocorals, respectively, with the dilution in carbon content being considered a fundamental aspect to improve cultivability in the latter study. In our study, most of the 23 isolated genera, spanning 16 families, belong to the two phyla (*Pseudomonadota* and *Bacteroidota*) that were also the most dominant in the cultivation-independent approach. These two phyla account for two-thirds of all ASVs, while the remaining portion is distributed between 13 unculturable, yet less represented phyla. Within the bacterial orders represented by cultivated bacteria, our isolates matched over one third of all ASVs detected in algal samples (35/106) with > 99% gene identity. Interestingly, 41 cultivated bacteria were not detected using cultivation-independent procedures, half of which not even in the seawater samples. These include all *Bacillota* and *Leucothrix* isolates, one *Taeseokella* isolate, and three clusters of *Flavobacteriaceae*. These results highlight the complementarity of the two approaches and how thorough the cultivation effort was, capturing strains that, either due to low abundance in the microbiome or primer inefficiency, were not detected in the amplicon sequencing dataset.

The bacterial culture collection included auxin-producing isolates (27% of the total) across all developmental stages. All auxin producers belonged to the phylum *Pseudomonadota* and included nine different genera and six clusters of isolates classified only to the family level. While IAA production was previously reported in several genera (*Sulfitobacter* (Amin et al., 2015), *Erythrobacter* (Zhang B. et al., 2022), *Alteromonas* (Bertrand, 2022), *Pseudoalteromonas* (Bertrand, 2022), and *Marinobacter* (Matthews et al., 2023)), to our knowledge, this is the first report in *Roseo*var*ius*, *Sphingorhabdus*, *Maricaulis,* and *Leucothrix* strains. The presence of this trait across a wide range of *Porphyra*-associated bacteria with differing abundances and temporal distributions suggests that it plays a significant role in regulating microbiome diversity and algal development. Besides promoting growth and development in the host (Basu et al., 2002; Kai et al., 2006; Klämbt et al., 1992), regulating reproduction (Uji and Mizuta, 2022), cell division and enlargement, photosynthesis, and the synthesis of bioactive compounds (Tan et al., 2021), IAA also serves as a signaling molecule in the holobiont (Cheng et al., 2023; Duca et al., 2014). However, at high concentrations, IAA can otherwise inhibit growth and disrupt the host’s homeostasis, a mechanism used by pathogenic bacteria (Duca et al., 2014; Spaepen and Vanderleyden, 2011). These negative effects are often associated with concentrations over 20 ppm (Cheng et al., 2023; Yokoya and Handro, 1996), while values below this threshold have been reported as beneficial to most macroalgae (Cheng et al., 2023; Kai et al., 2006; Yokoya and Handro, 1996), including *Porphyra* (De-lin et al., 1995; Lin and Stekoll, 2007). Although IAA concentrations measured at the laboratory scale may not translate to real-world scenarios in aquaculture production, some bacteria surpassed the 20-ppm margin, most notably the *Alteromonas* isolated from *P. dioica* at stage S3. Having been extensively associated with algicidal activity (Abdul Malik et al., 2020; Goecke et al., 2010; Hollants et al., 2013; Ward et al., 2020), these bacteria could indeed be harmful to *Porphyra* through the excess production of IAA. Nevertheless, the remaining isolates hold great potential as beneficial bacteria, especially those that achieved IAA concentrations between 10 and 20 ppm (*Leucothrix*, *Sulfitobacter*, *Roseovarius*, *Marinobacter,* and *Erythrobacter*). Most of them have also been reported to exhibit other complementary bioactivities, from the production of B vitamins to antimicrobial agents (Alsufyani et al., 2020; Amin et al., 2015; Bertrand, 2022; Burgunter-Delamare et al., 2024; Hosseini et al., 2024; Sultana et al., 2023; Tang et al., 2019; Tareen et al., 2022). According to the cultivation-independent data, *Sulfitobacter* and *Marinobacter* are two genera of considerable relative abundance in the prokaryotic communities of *Porphyra* for which IAA production was observed, which suggests their association with the algae is particularly important under the studied circumstances.

Testing how IAA biosynthesis is influenced in co-cultivation with the algal host and how it affects the holobiont will be of great value to better understand the roles played by these bacteria in algal growth promotion. This, coupled with the screening for other bioactivities, such as the production of the essential vitamin B12, antimicrobial, or antialgal agents, will help delineate their likely behavior as beneficial or pathogenic symbionts and how they should be handled in future microbiome engineering attempts.

### 4.4 Concluding remarks

This study shows that the early developmental stages of Atlantic Nori harbor diverse and likely dynamic prokaryotic communities, composed of both culturable and so-far unculturable taxa that likely establish a spectrum of associations with their algal hosts. Taken together, our observations indicate that the microbiome of farmed *Porphyra* may function less as a collection of isolated species and more as an interactive assemblage of overlapping as well as complementary traits, as hypothesized in other studies (Wang et al., 2022), the expression of which likely varies with host developmental stage and environmental context. Importantly, a more comprehensive understanding of microbiome-assisted algal development and health awaits “all-encompassing” studies that integrate insights beyond bacteria, incorporating pivotal players such as viruses (eukaryotic and prokaryotic) and microeukaryotes including fungi, protozoa, and microalgae. Harnessing this microbial functional diversity presents a tangible opportunity for sustainable aquaculture. By identifying and combining key symbionts with complementary roles (e.g., growth promoters, morphogenesis inducers, nutrient cyclers, and antagonists of pathogens) it may be possible to guide microbiome manipulation toward stable, beneficial states. Such steering could be achieved through targeted inoculation strategies, selective enrichment of desirable strains, or manipulation of cultivation conditions that favor beneficial taxa while suppressing opportunistic pathogens. Ultimately, this knowledge provides a roadmap for microbiome engineering approaches aimed at ensuring disease-free, resilient, and productive *Porphyra* cultivation systems.

## Data Availability

All sequencing data generated in this study are publicly available in the NCBI database (https://www.ncbi.nlm.nih.gov/). The 16S rRNA gene amplicon sequencing data, obtained using Illumina MiSeq technology, are available under the BioProject accession number PRJNA1309074 (Sample accession numbers: SAMN50730945 – SAMN50730980; Run accession numbers: SRR35071199 – SRR35071234). The 16S rRNA gene sequences of cultured bacterial isolates, obtained via Sanger sequencing, are available in GenBank under the accession numbers PX218965-PX219098.

## Footnotes

1
https://www.algaplus.pt/

2
https://www.stabvida.com/pt

## Supplementary material

Supplementary Figure S1 Macroscopic and microscopic pictures of farmed *Porphyra* species at the three nursery developmental stages. **(A)** Stage S1–conchocelis (sporophyte, 2N); **(B)** stage S2–conchosporangia (sporophyte, 2N); and **(C)** stage S3–young blades (gametophyte, N). The upper row shows macroscopic views of the algae within 50 mL Falcon tubes, while the lower row presents corresponding microscopic images of each developmental stage.

Supplementary Figure S2 Rarefaction curves. Curves in the graph display the relationship between sequencing depth (number of reads, x axis) and Observed ASVs (y axis) for **(A)** all samples and **(B)** only the algal tissue samples analyzed in this study. Curves’ plateaus were evaluated for choosing a rarefaction threshold (941reads) in our study to assess alpha diversity, as displayed in Figure 2. Pdi–*Porphyra dioica*; Pu–*Porphyra umbilicalis*; W–culturing water; 1,2,3–developmental stages S1, S2 and S3; a,b,c,d–independent replicate samples.

Supplementary Figure S3 Additional *Porphyra* and water-associated bacterial genera differently represented across developmental stages. Box-and-whisker plots showing the relative abundance distributions of those bacterial genera with abundance >0.5% and not depicted in Figure 4. Legend is reported for each plot (Pdi for *P. dioica*; Pu for *P. umbilicalis*; W for water; numbers 1-3 represent developmental stages). The central box represents the distance between the 25th and 75th percentiles. The median is marked with a black line. Whiskers identify the 10th and 90th percentiles. Kruskal-Wallis test controlled for multiple testing using false discovery rate (FDR); p-values, when significant, are printed below the corresponding plot.

Supplementary Figure S4 16S rRNA gene-based phylogenetic tree of the order *Alteromonadales*. Phylogenetic tree of *Porphyra*-derived bacterial isolates from the order *Alteromonadales* (*N* = 44) and their closest type strains along with ASVs from this study with over 99% sequence identity to isolates (in bold). The isolates are identified with their accession number and strain ID (Pdi–isolated from *Porphyra dioica*; Pu–isolated from *P. umbilicalis*), and their taxonomy classification according to Silva, with eventual manual curation of the nomenclature according to the LPSN. The tree was constructed using the maximum likelihood method (highest log-likelihood of -2,256.83) with 1,000 bootstrap repetitions, applying the Kimura 2-parameter model (Kimura, 1980) with the inclusion of a rate variation model (+I, 35.28% sites) to allow for evolutionary invariable sites (K2+I). The branch lengths are measured in the number of substitutions per site, with the tree drawn to scale. All positions with less than 85% site coverage were eliminated, and ambiguous bases were allowed at any position using partial deletion. Bootstrap values are shown (black circle) at each branch to indicate the level of statistical support. An outgroup with 7 *Roseobacter* sp. sequences was used to root the tree. Clusters in green contain sequences with no corresponding ASVs.

Supplementary Figure S5 16S rRNA gene-based phylogenetic tree of the order *Rhodobacterales*. Phylogenetic tree of bacterial isolates from the order *Rhodobacterales* (*N* = 35) and their closest type strains (TS) along with ASVs with over 99% sequence identity (in bold). The isolates are identified with their isolate number (Pdi – isolated from *Porphyra dioica*; Pu – isolated from *P. umbilicalis*), and their taxonomy classification according to Silva, with eventual manual corrections made to update the nomenclature following LPSN. The tree was constructed using the maximum likelihood method (highest log-likelihood of -3,945.34) with 1,000 bootstrap repetitions, applying the General Time Reversible model (Nei and Kumar, 2000) employing discrete Gamma distributions (5 categories, +G, parameter = 0.1634) to account for evolutionary rate variation across sites and the inclusion of a rate variation model (+I, 40.04% sites) to allow for evolutionarily invariable sites (GTR+G+I). The branch lengths are measured in the number of substitutions per site, with the tree drawn to scale. All positions with less than 85% site coverage were eliminated, and ambiguous bases were allowed at any position using partial deletion. Bootstrap values are shown at each branch to indicate the level of statistical support. An outgroup with 6 *Agrobacterium* sequences was used to root the tree. Clusters colored in green contain sequences with no corresponding ASVs, while clusters colored in blue contain sequences that may represent novel taxa.

Supplementary Figure S6 16S rRNA gene-based phylogenetic tree of the order *Flavobacteriales*. Phylogenetic tree of bacterial isolates from the order *Flavobacteriales* (*N* = 17) and their closest type strains (TS) along with ASVs with over 99% sequence identity (in bold). The isolates are identified with their isolate number (Pdi – isolated from *Porphyra dioica*; Pu – isolated from *P. umbilicalis*), and their taxonomy classification according to Silva, with eventual manual corrections made to update the nomenclature following LPSN. The tree was constructed using the maximum likelihood method (highest log-likelihood of -871.46) with 1,000 bootstrap repetitions, applying the Kimura 2-parameter model (Kimura, 1980) with the inclusion of a rate variation model (+I, 33.41% sites) to allow for evolutionary invariable sites (K2+I). The branch lengths are measured in the number of substitutions per site, with the tree drawn to scale. All positions with less than 85% site coverage were eliminated, and ambiguous bases were allowed at any position using partial deletion. Bootstrap values are shown at each branch to indicate the level of statistical support. An outgroup with four *Algoriphagus* sequences was used to root the tree. Clusters colored in green contain sequences with no corresponding ASVs, while clusters colored in blue contain sequences that may represent novel taxa.
