# Supplementary material for: Prokaryotic community structure and auxin biosynthesis in early developmental stages of farmed Atlantic Nori (Porphyra spp.)
Source: Front Microbiol. 2026 Jan 21;16:1750184. doi: 10.3389/fmicb.2025.1750184 (PMC12868259; doi:10.3389/fmicb.2025.1750184)
Supplement: Supplementary file 2 [file Data_Sheet_2.zip › Supplementary Material Presentation/Suppl Fig S3.pdf]

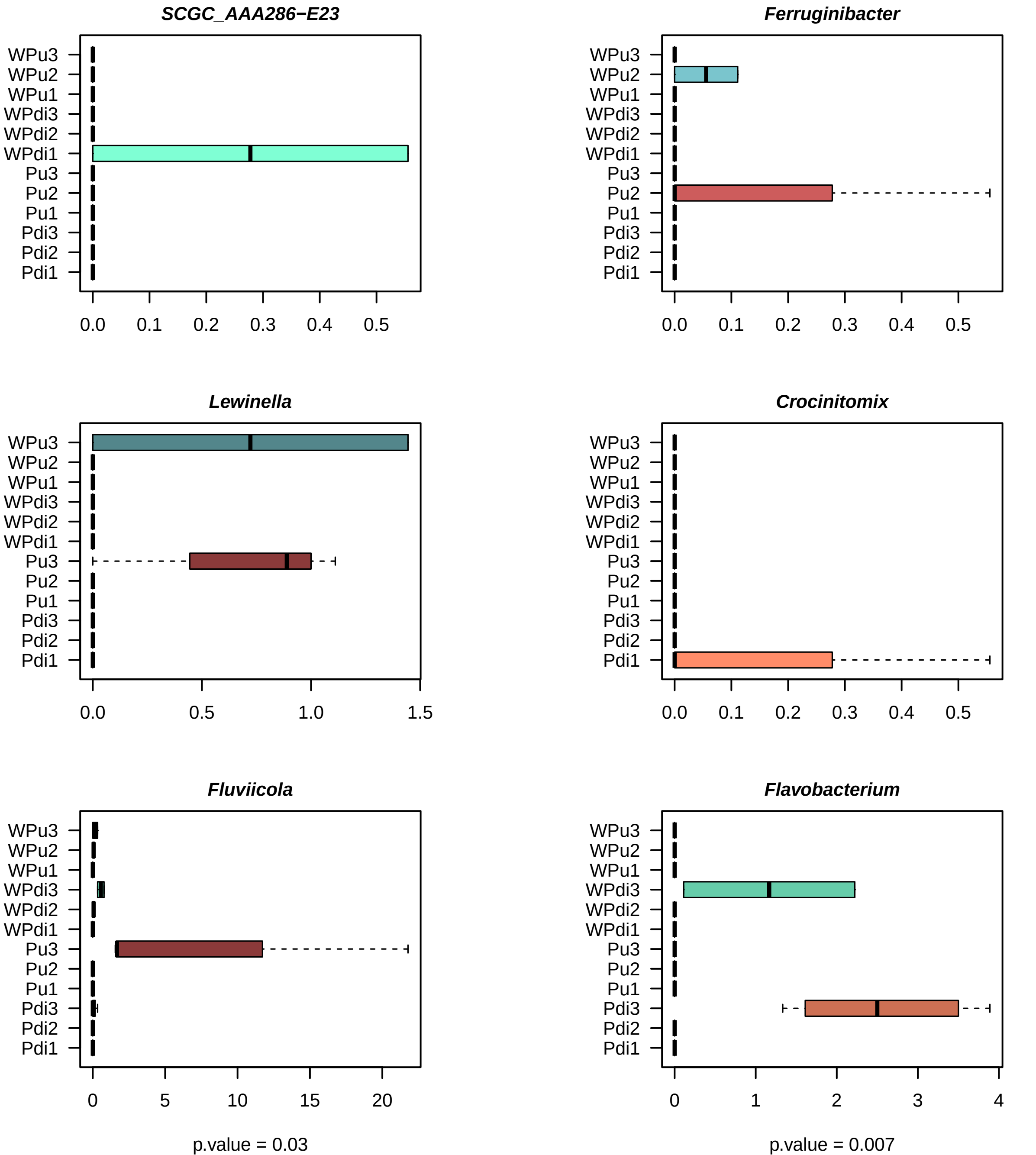

SCGC_AAA286−E23
Ferruginibacter
Lewinella
Crocinitomix
Fluviicola
Flavobacterium
WPu3
WPu2
WPu1
WPdi3
WPdi2
WPdi1
Pu3
Pu2
Pu1
Pdi3
Pdi2
Pdi1
0.0
0.1
0.2
0.3
0.4
0.5
0.0
0.5
1.0
1.5
0
5
10
15
20
0
1
2
3
4
p.value = 0.03
p.value = 0.007

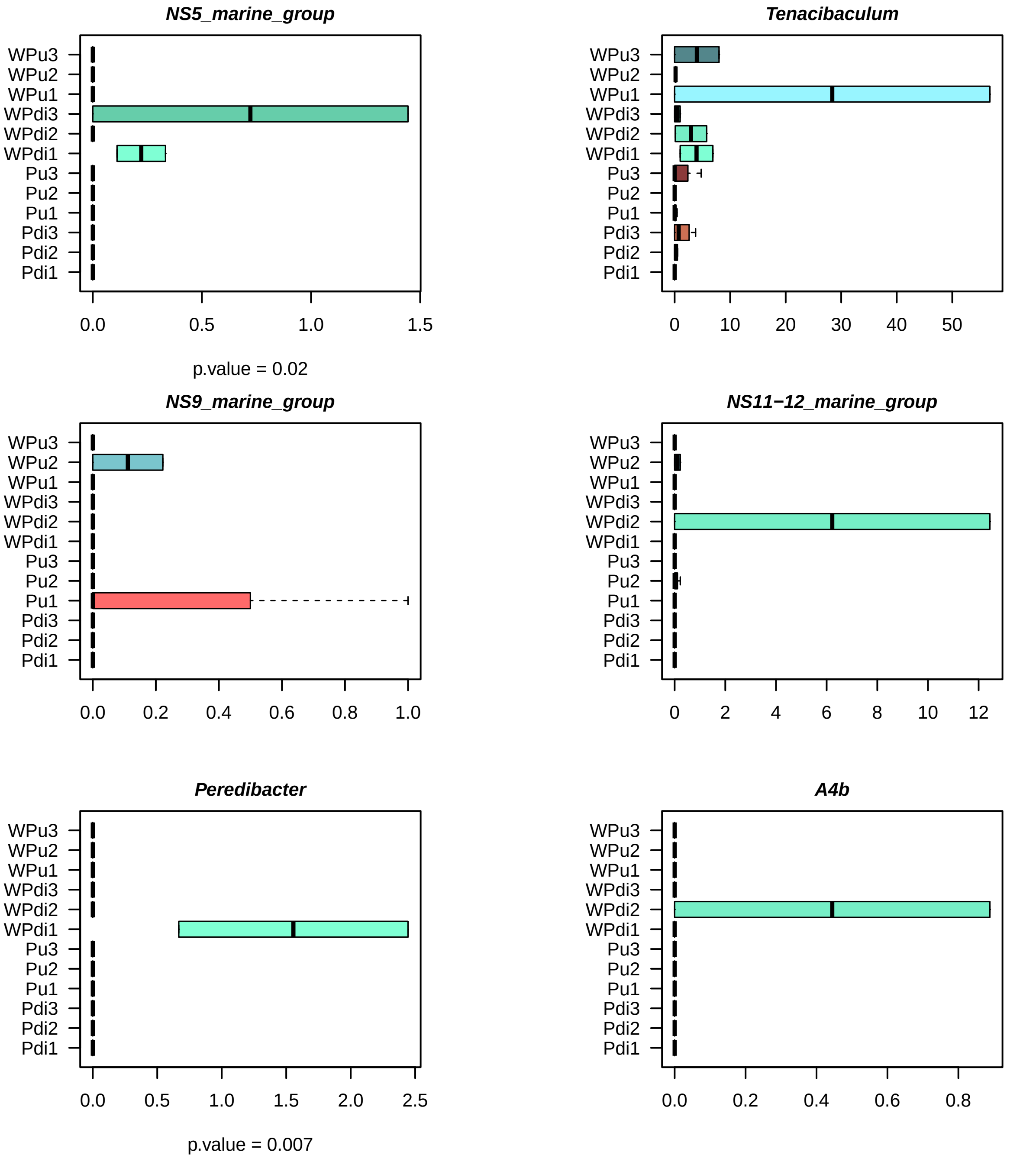
NS5_marine_group
Tenacibaculum
NS9_marine_group
NS11−12_marine_group
Peredibacter
A4b
WPu3
WPu2
WPu1
WPdi3
WPdi2
WPdi1
Pu3
Pu2
Pu1
Pdi3
Pdi2
Pdi1
0.0 0.5 1.0 1.5
0 10 20 30 40 50
0.0 0.2 0.4 0.6 0.8 1.0
0 2 4 6 8 10 12
0.0 0.5 1.0 1.5 2.0 2.5
0.0 0.2 0.4 0.6 0.8
p.value = 0.02
p.value = 0.007

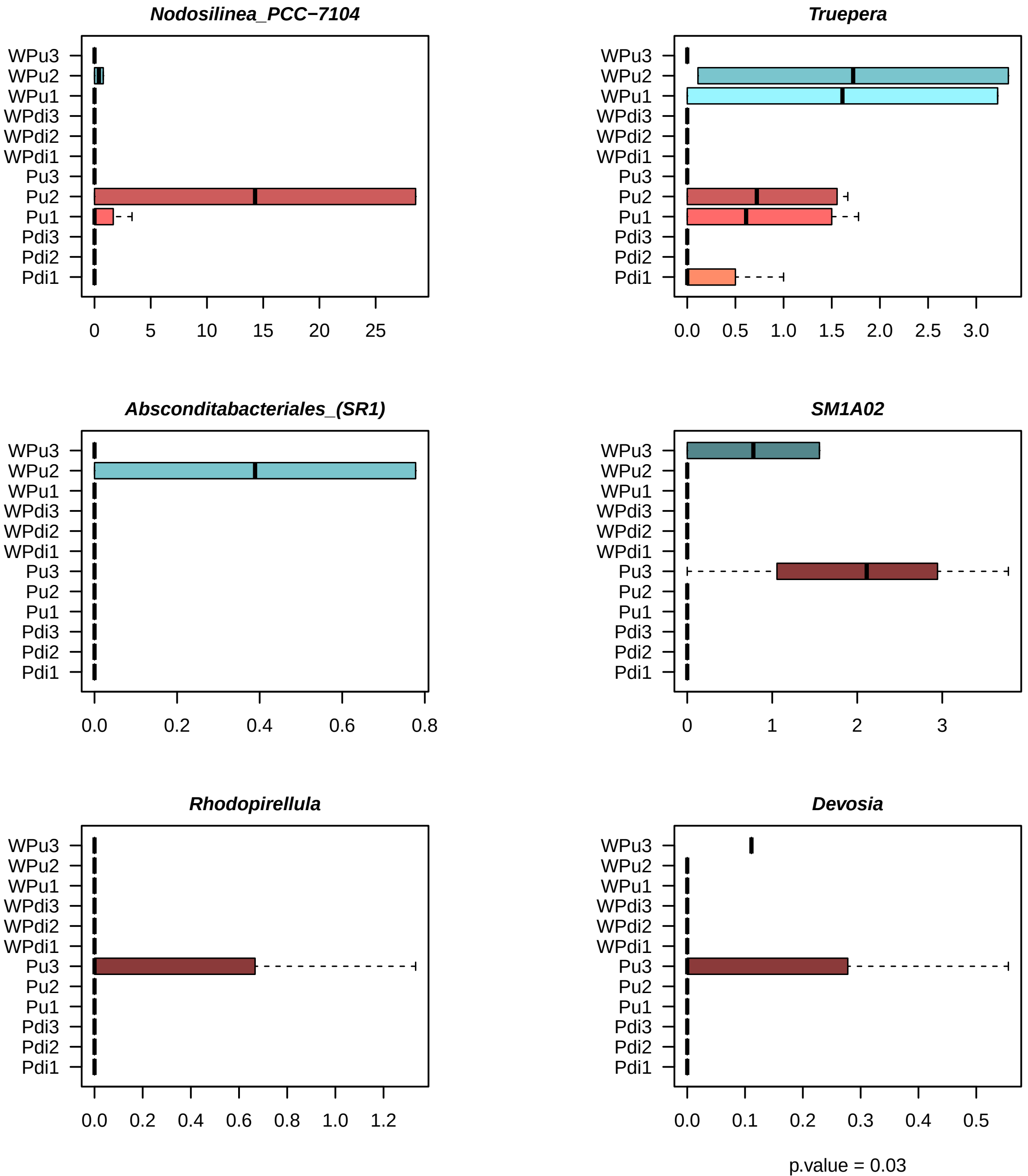
Nodosilinea_PCC−7104
Truepera
Absconditabacteriales_(SR1)
SM1A02
Rhodopirellula
Devosia
WPu3
WPu2
WPu1
WPdi3
WPdi2
WPdi1
Pu3
Pu2
Pu1
Pdi3
Pdi2
Pdi1
0 5 10 15 20 25
0.0 0.5 1.0 1.5 2.0 2.5 3.0
0.0 0.2 0.4 0.6 0.8
0 1 2 3
0.0 0.2 0.4 0.6 0.8 1.0 1.2
0.0 0.1 0.2 0.3 0.4 0.5
p.value = 0.03

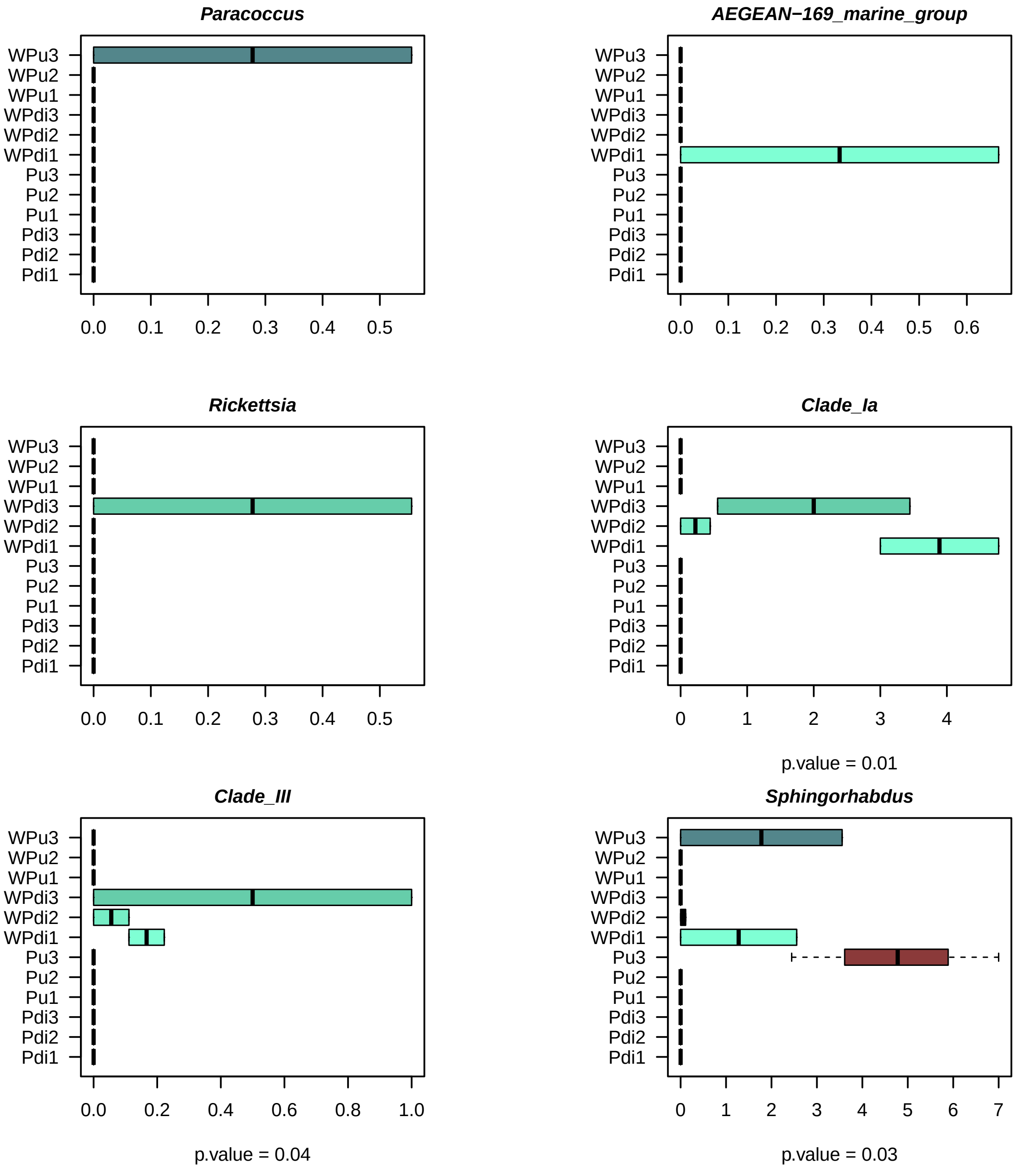

Paracoccus
AEGEAN−169_marine_group
Rickettsia
Clade_Ia
Clade_III
Sphingorhabdus
WPu3
WPu2
WPu1
WPdi3
WPdi2
WPdi1
Pu3
Pu2
Pu1
Pdi3
Pdi2
Pdi1
0.0 0.1 0.2 0.3 0.4 0.5
0.0 0.1 0.2 0.3 0.4 0.5 0.6
0.0 0.1 0.2 0.3 0.4 0.5
0 1 2 3 4
p.value = 0.01
0.0 0.2 0.4 0.6 0.8 1.0
p.value = 0.04
0 1 2 3 4 5 6 7
p.value = 0.03

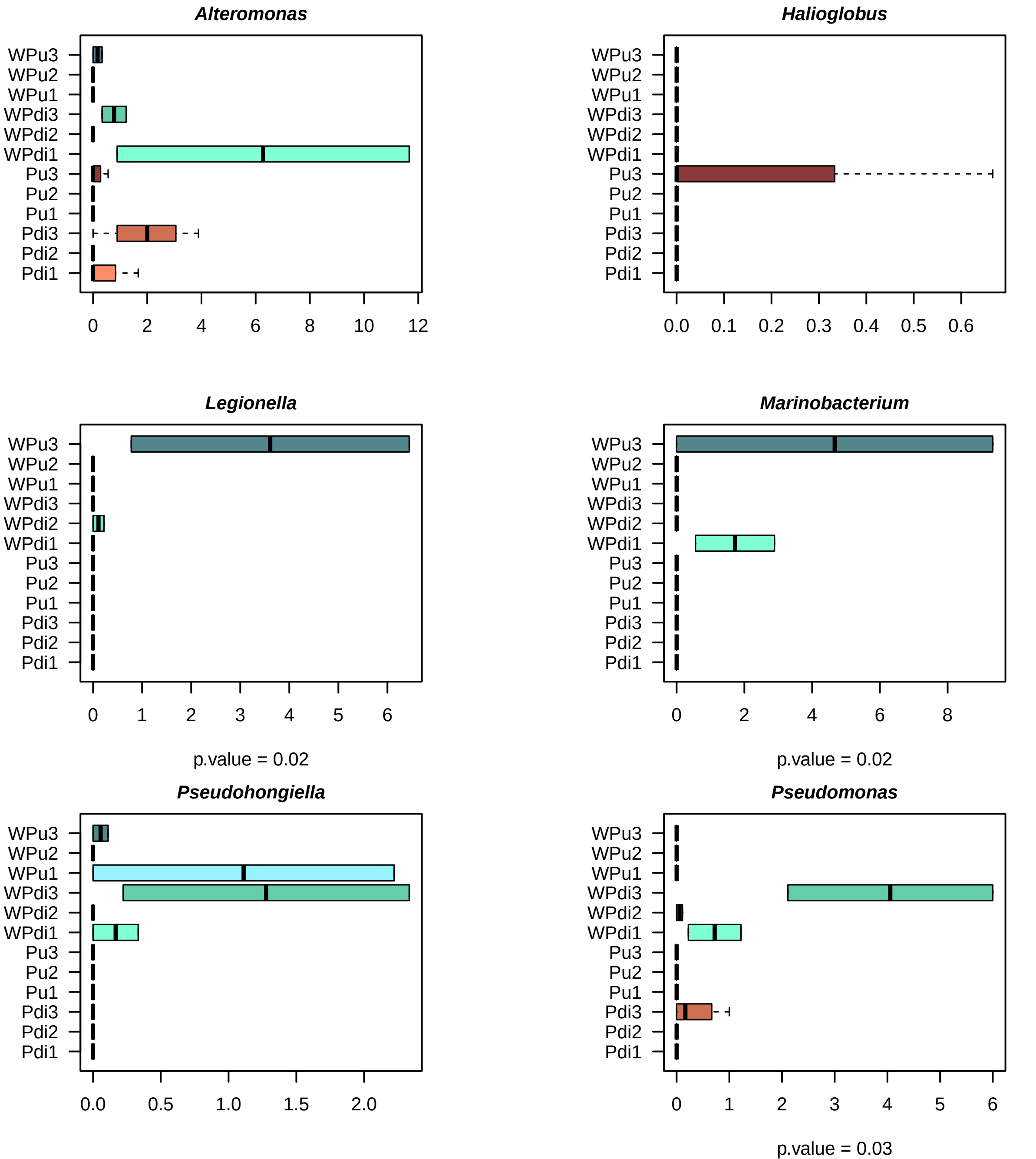

Alteromonas
WPu3
WPu2
WPu1
WPdi3
WPdi2
WPdi1
Pu3
Pu2
Pu1
Pdi3
Pdi2
Pdi1
0 2 4 6 8 10 12
Halioglobus
0.0 0.1 0.2 0.3 0.4 0.5 0.6
Legionella
0 1 2 3 4 5 6
p.value = 0.02
Marinobacterium
0 2 4 6 8
p.value = 0.02
Pseudohongiella
0.0 0.5 1.0 1.5 2.0
Pseudomonas
0 1 2 3 4 5 6
p.value = 0.03

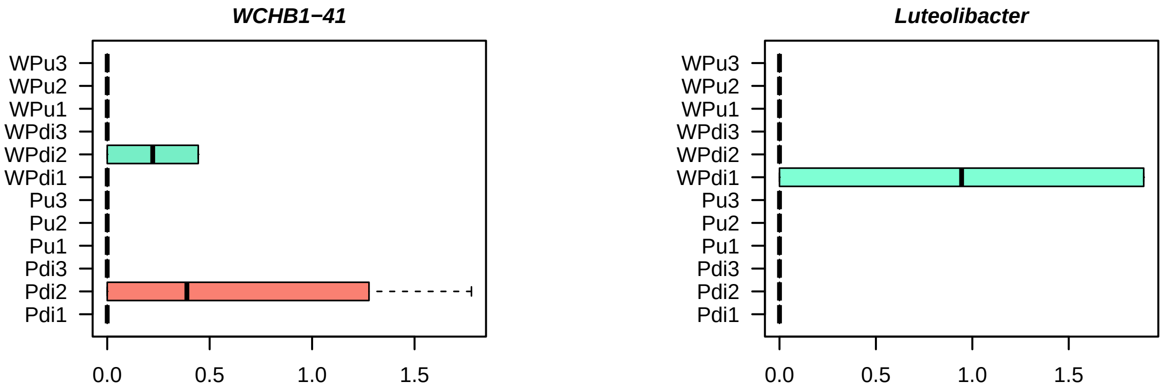
WCHB1−41
WPu3
WPu2
WPu1
WPdi3
WPdi2
WPdi1
Pu3
Pu2
Pu1
Pdi3
Pdi2
Pdi1
0.0
0.5
1.0
1.5
Luteolibacter
WPu3
WPu2
WPu1
WPdi3
WPdi2
WPdi1
Pu3
Pu2
Pu1
Pdi3
Pdi2
Pdi1
0.0
0.5
1.0
1.5
