# Supplementary figures and images for: Prokaryotic community structure and auxin biosynthesis in early developmental stages of farmed Atlantic Nori (Porphyra spp.)
Source: Front Microbiol. 2026 Jan 21;16:1750184. doi: 10.3389/fmicb.2025.1750184 (PMC12868259; doi:10.3389/fmicb.2025.1750184)

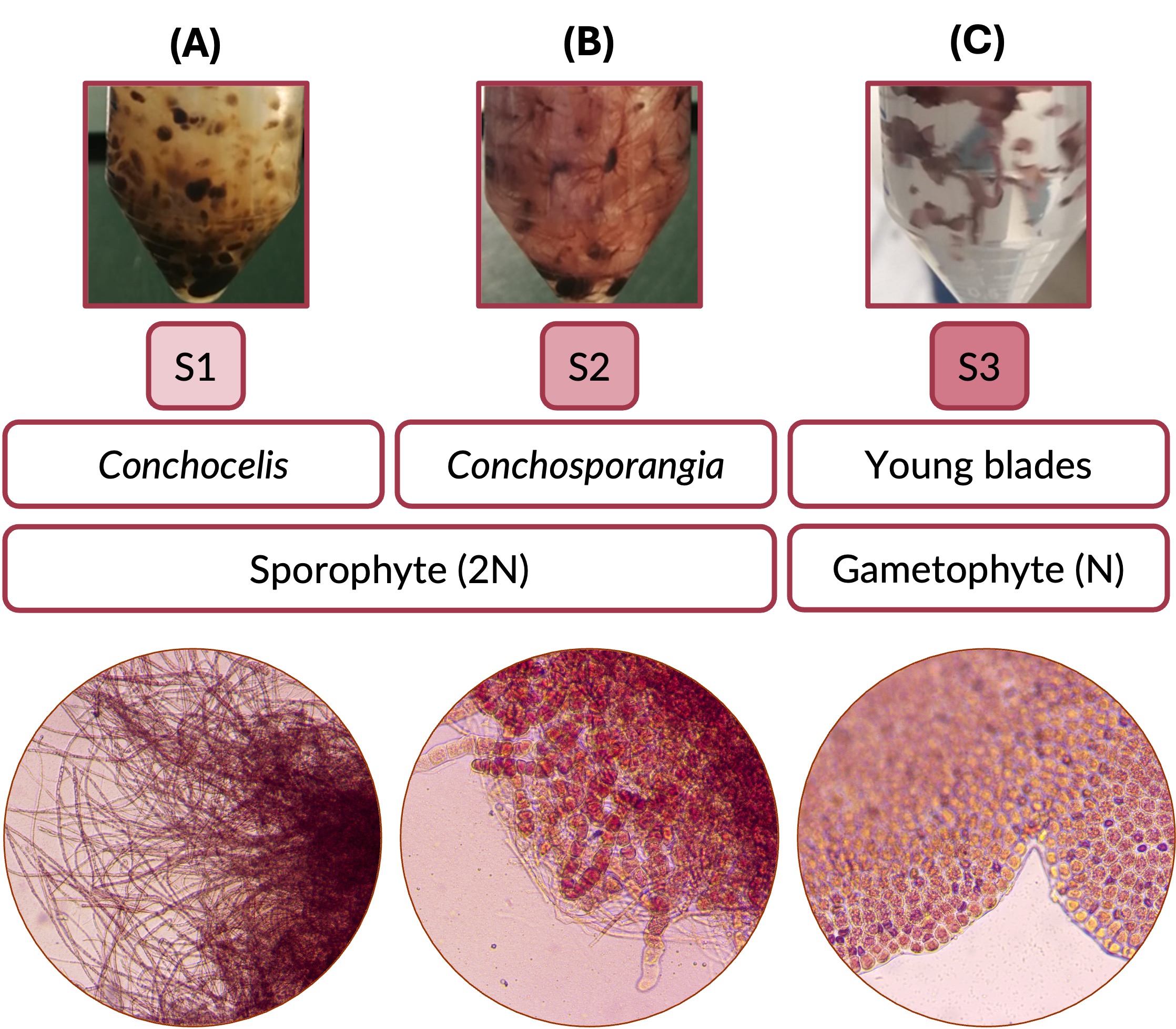

Supplement: Supplementary file 2 [file Data_Sheet_2.zip › Supplementary Material Presentation/Suppl Fig S1.jpg]

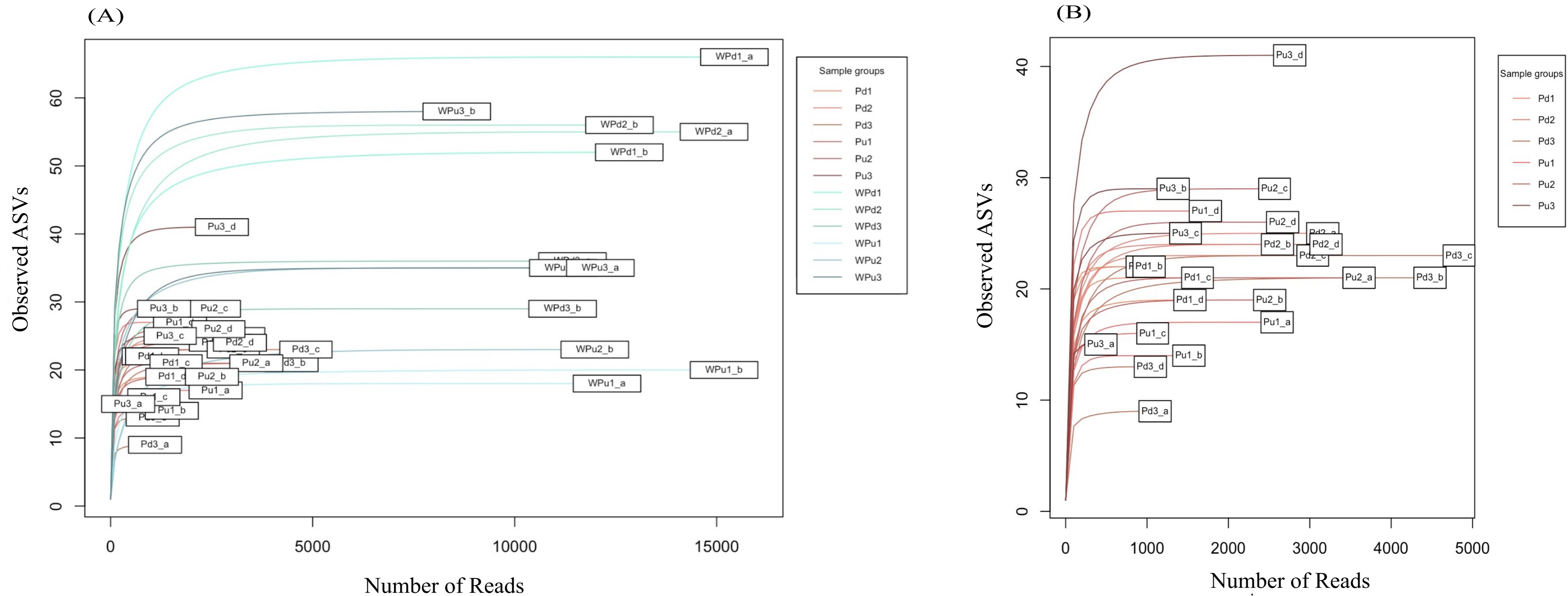

Supplement: Supplementary file 2 [file Data_Sheet_2.zip › Supplementary Material Presentation/Suppl Fig S2.pdf]

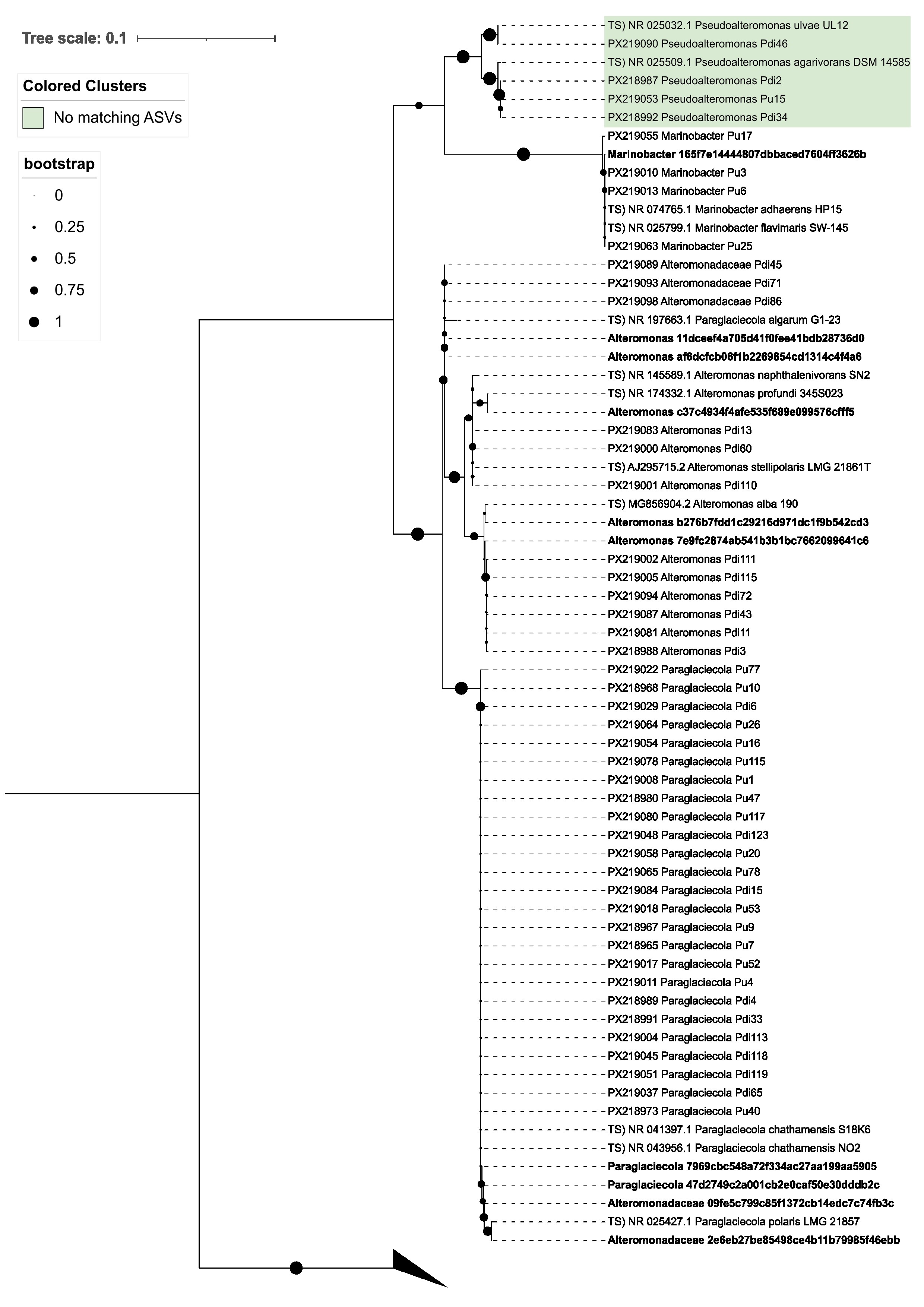

Supplement: Supplementary file 2 [file Data_Sheet_2.zip › Supplementary Material Presentation/Suppl Fig S4.jpg]

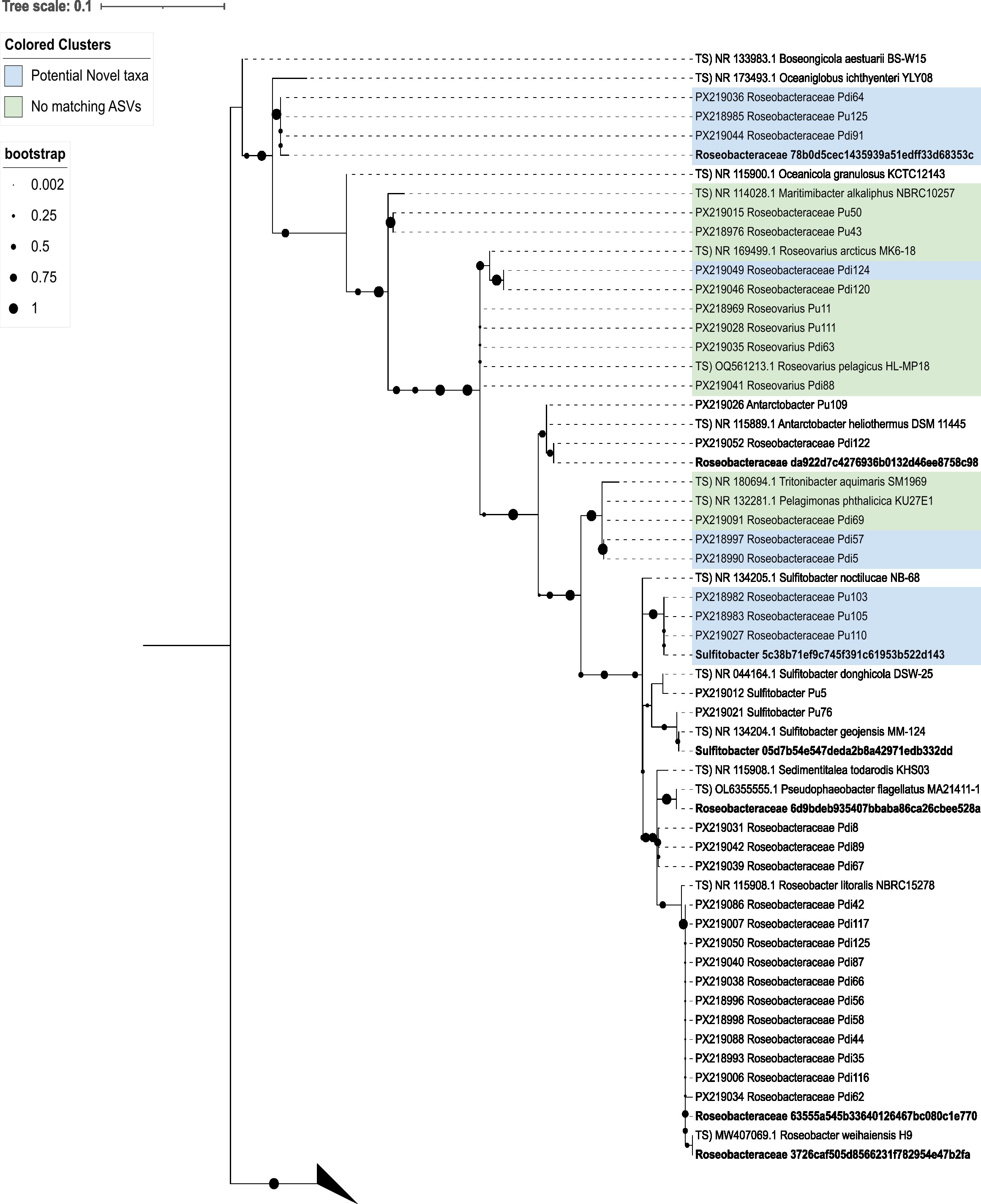

Supplement: Supplementary file 2 [file Data_Sheet_2.zip › Supplementary Material Presentation/Suppl Fig S5.jpg]

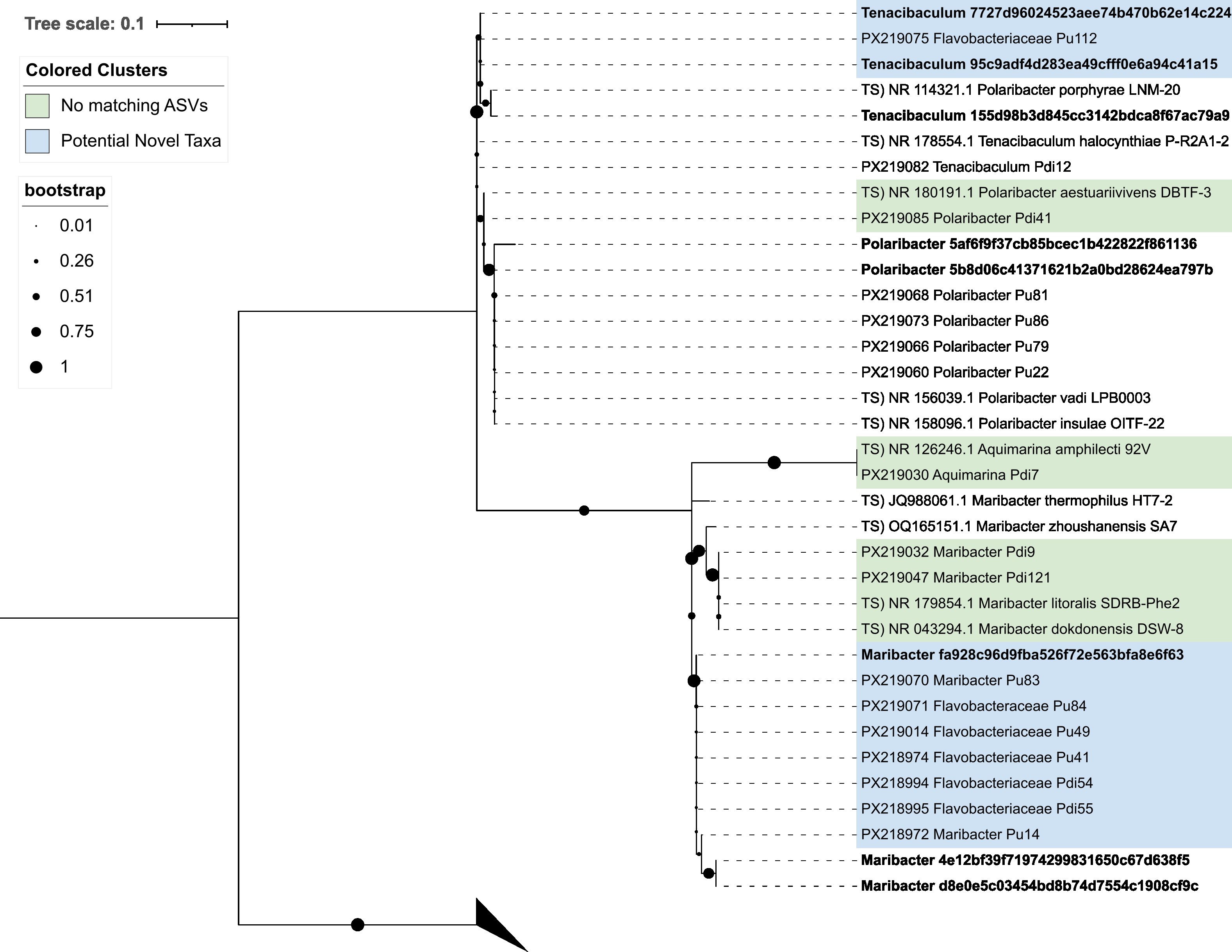

Supplement: Supplementary file 2 [file Data_Sheet_2.zip › Supplementary Material Presentation/Suppl Fig S6.jpg]
